# Association of Gene Polymorphisms with Normal Tension Glaucoma: A Systematic Review and Meta-Analysis

**DOI:** 10.3390/genes15040491

**Published:** 2024-04-14

**Authors:** Lijie Pan, Jian Wu, Ningli Wang

**Affiliations:** 1 Beijing Institute of Ophthalmology, Beijing Tongren Eye Center, Beijing Tongren Hospital, Capital Medical University, Beijing Key Laboratory of Ophthalmology and Visual Sciences, No. 1 Dong Jiao Min Xiang Street, Dongcheng District, Beijing 100730, China; plj2761@163.com; 2 School of Life Sciences, Peking University, No. 5 Yiheyuan Road, Haidian District, Beijing 100871, China; 3 Henan Academy of Innovations in Medical Science, No. 2 Biotechnology Street, Hangkonggang District, Zhengzhou 450000, China

**Keywords:** NTG, single nucleotide polymorphism, genetic polymorphism, meta-analysis

## Abstract

Background: Normal tension glaucoma (NTG) is becoming a more and more serious problem, especially in Asia. But the pathological mechanisms are still not illustrated clearly. We carried out this research to uncover the gene polymorphisms with NTG. Methods: We searched in Web of Science, Embase, Pubmed and Cochrane databases for qualified case-control studies investigating the association between single nucleotide polymorphisms (SNPs) and NTG risk. Odds ratios (ORs) and 95% confidence intervals (CIs) for each SNP were estimated by fixed- or random-effect models. Sensitivity analysis was also performed to strengthen the reliability of the results. Results: Fifty-six studies involving 33 candidate SNPs in 14 genetic loci were verified to be eligible for our meta-analysis. Significant associations were found between 16 SNPs (rs166850 of *OPA1*; rs10451941 of *OPA1*; rs735860 of *ELOVL5*; rs678350 of *HK2*; c.603T>A/Met98Lys of *OPTN*; c.412G>A/Thr34Thr of *OPTN*; rs10759930 of *TLR4*; rs1927914 of *TLR4*; rs1927911 of *TLR4*; c.*70C>G of *EDNRA*; rs1042522/-Arg72Pro of *P53*; rs10483727 of *SIX1-SIX6*; rs33912345 of *SIX1-SIX6*; rs2033008 of *NCK2*; rs3213787 of *SRBD1* and c.231G>A of *EDNRA*) with increased or decreased risk of NTG. Conclusions: In this study, we confirmed 16 genetic polymorphisms in 10 genes (*OPA1*, *ELOVL5*, *HK2*, *OPTN*, *TLR4*, *EDNRA*, *P53*, *NCK2*, *SRBD1* and *SIX1-SIX6*) were associated with NTG.

## 1. Introduction

Glaucoma is a disease characterized by optic neuropathy with the symptoms of visual impairment and visual field loss. It is usually associated with an increase in intraocular pressure (IOP) [1]. Normal-tension glaucoma (NTG) is always supposed to be a spectrum of primary open-angle glaucoma (POAG) [2,3] but with an IOP in the normal range [4], featured by normal anterior chamber depth, retinal nerve fiber layer (RNFL) thinning and progressing optic neuropathy [5]. NTG is becoming a more and more serious problem, with especially high prevalence in Asia. The morbidity of POAG in East Asians is from 1–4% [6], of which NTG contributes up to 95% [7]. However, it is reported that European Caucasians suffer less from NTG, which takes up about one-third of POAG patients [6]. It is plausible for us to suggest the incidence of NTG differs among various ethnicities. What is more, it should be noted that with the increased longevity, the incidence of NTG is likely to rise.

The pathological mechanisms of NTG are still not illustrated clearly and may be ascribed to multiple factors. Some hypotheses related to the pathogenesis include cardiovascular and neurovascular diseases, vasospasm, oxidative stress, endothelial dysfunction and abnormal biomechanics of the lamina cribrosa and so on [6,8]. Genetic polymorphism is supposed to play an important role in NTG. For one reason, people could suffer from glaucoma at different ages, and genetic predisposition may mean an earlier onset [9]. For another, gene detection has come into effect in the recognition of allele mutations, especially for young Mendelian glaucoma [10]. Some genes have been found to be associated with NTG, including Optineurin (*OPTN*), TANK-binding kinase (*TBK1*) and Myocilin (*MYOC*) [10]. 

In recent years, more interest has been attracted to the topic of the association between gene polymorphisms and NTG. Many studies have pointed out the relationship and statistical significance of gene mutations in the disease [11,12]. However, it confuses us that the former research studies differ from each other in involved SNPs and statistical significance influenced by different study areas, population ethnicity and research heterogeneity. 

Our meta-analysis aims to collect and summarize all the satisfactory literature, and analyze the effect of allele mutations and gene functions specific to the onset of NTG, so as to provide an extensive exploration and evidence for us to uncover the gene polymorphisms with NTG.

## 2. Materials and Methods

The research protocol has been registered in PROSPERO with the ID CRD42022326782.

### 2.1. Search Strategy

We conducted the literature search and selection mainly from the following four databases: Web of Science, Embase, Pubmed and Cochrane. Three groups of MeSH terms were put into the search interface to frame the Boolean search strategy as follows, “(((Genes[MeSH Terms]) OR ((((((((Genes[Title/Abstract]) OR (Gene[Title/Abstract])) OR (Cistron[Title/Abstract])) OR (Cistrons[Title/Abstract])) OR (Genetic Materials[Title/Abstract])) OR (Genetic Material[Title/Abstract])) OR (Material, Genetic[Title/Abstract])) OR (Materials, Genetic[Title/Abstract]))) OR ((Polymorphism, Single Nucleotide[MeSH Terms]) OR (((((((Polymorphism, Single Nucleotide[Title/Abstract]) OR (Nucleotide Polymorphism, Single[Title/Abstract])) OR (Nucleotide Polymorphisms, Single[Title/Abstract])) OR (Polymorphisms, Single Nucleotide[Title/Abstract])) OR (Single Nucleotide Polymorphisms[Title/Abstract])) OR (SNPs[Title/Abstract])) OR (Single Nucleotide Polymorphism[Title/Abstract])))) AND ((“Low Tension Glaucoma”[Mesh]) OR ((((((Low Tension Glaucoma[Title/Abstract]) OR (Glaucoma, Low Tension[Title/Abstract])) OR (Low Tension Glaucomas[Title/Abstract])) OR (Normal Tension Glaucoma[Title/Abstract])) OR (Glaucoma, Normal Tension[Title/Abstract])) OR (Normal Tension Glaucomas[Title/Abstract])))”. In this way, a systematic retrospect of original articles of all types analyzing the association between gene polymorphisms and NTG risk was acquired.

#### 2.1.1. Inclusion Criteria

(1) The diagnostic standard of NTG should be indicated clearly;
(2) Cohort studies involving NTG patients and healthy controls which evaluate the potential association of specific gene mutations, SNPs, allele variations related to pathogenesis of the disease;
(3) Some important information should be included: demographic features such as age and sex, allele or genotype frequencies of SNPs in both case and control groups, index of association strength such as odds ratio (OR) with 95% confidence interval (CI).

#### 2.1.2. Exclusion Criteria

(1) Studies published in the form of meta-analysis, review, case report, patent, guideline, conference abstract and book chapters;
(2) Studied objects are animals;
(3) Studies not written in English;
(4) Studies which lack OR value, only refers to POAG but not NTG or did not indicate a clear definition of POAG.

The included studies were come to by agreement of all the contributors of this article. 

### 2.2. Data Extraction

Two reviewers independently screened and searched for the needed data from all the eligible literature. Disparities were discussed and solved by all the reviewers until consensus were reached. The following data were extracted and recollected in the table: reference (first author, year of publication), involved ethnicity, sample size of both case and control groups, demographic features including age and sex of two groups and genotyping method. If the basic or allele data of NTG were reported together with high-tension glaucoma (HTG) in POAG, we selected data specifically for NTG to document.

### 2.3. Quality Assessment

The methodological quality of all the eligible articles were assessed according to the Newcastle–Ottawa scale (NOS) [13]. There are three evaluation criteria in consideration: case selection, comparability and exposure. The quality of studies was recorded in the form of stars and the maximum star was 9. Studies acquired 6 stars or greater were considered up to our analyzing standard and qualified for further assessment.

### 2.4. Meta-Analysis

SNPs and gene mutations were qualified for meta-analysis if they were investigated by at least two studies. The statistical significance was recorded as OR [95% CI]. Allele frequency in eligible studies was calculated and screened after data organization, and minor allele for specific SNP was determined if it was consistent in all ethnic groups. Meta-analysis was processed by pooling OR values from eligible studies for the allele model (B versus A), dominant model (BB+ AB versus AA), recessive model (BB versus AA+ AB), heterozygote model (AB versus AA) and homozygote model (BB versus AA), respectively. Stata version 15.1 software (Stata Corporation, College Station, TX, USA) was used to perform statistical analyses. The difference was considered to be of statistical significance if the *p* value was less than 0.05.

The heterogeneity tests for independent studies orienting the same SNPs were conducted by means of Q test and *I*^2^ test. *p* value was used as testing statistics for Q test, and heterogeneity existed if it was below 0.05. Similarly, if that *I*^2^ value was greater than 50% it suggested a possibility of heterogeneity [14]. Then we chose fixed-effect model for studies without obvious heterogeneity to analyze the OR value for each gene polymorphism. On the contrary, random-effect model was chosen. What is more, Begg’s Test was used to evaluate the publication bias among included articles [15].

Subjects with NTG were further classified into different ethnicities and stratified meta-analysis was conducted for them. Sensitivity analysis was alco carried out.

## 3. Results

### 3.1. Selection of Qualified Literature 

**Figure 1:**
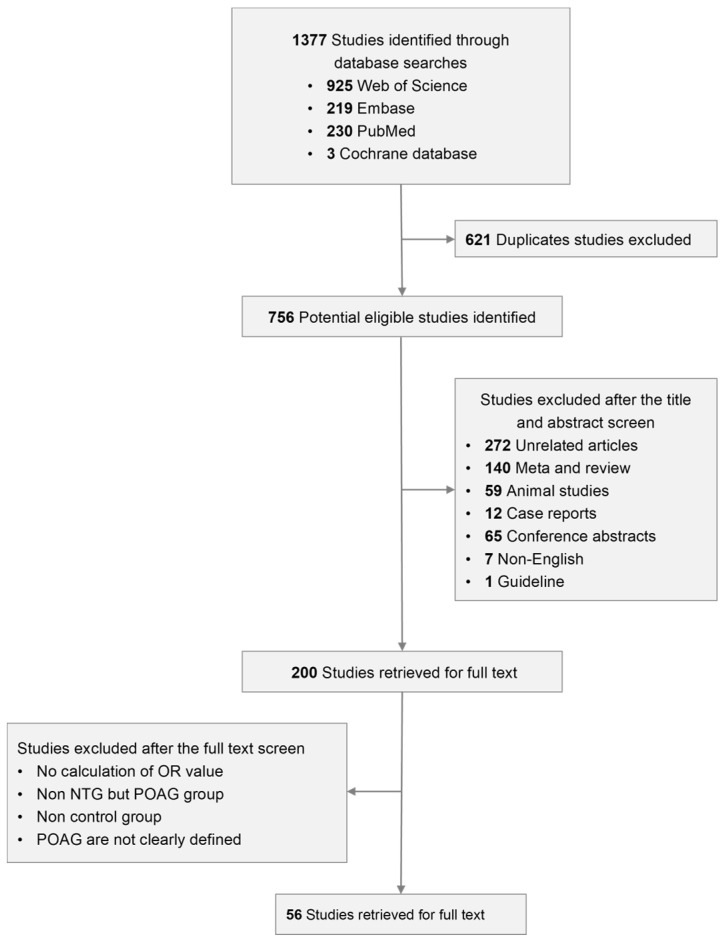
The procedure of literature selection for meta-analysis.

The procedure of our selection strategy can be acquired from Figure 1. 

A total of 1377 studies could be searched through the four databases, of which 925 were from Web of Science, 219 from Embase, 230 from Pubmed and the remaining 3 from Cochrane Database. Among them, 493 were duplicated articles which should be excluded. We then screened for the title as well as abstract of the other 621 studies and removed a large part of the literature, for there were 272 unrelated articles, 140 meta-analyses and reviews, 65 conference abstracts, 59 animal studies, 12 case reports, 7 non-English articles and 1 guideline. Two hundred studies were left for us to be read through and the articles were to be excluded if important information was absent such as if there was no calculation of the OR value, no NTG group but only POAG group, no control group or POAG was not defined clearly. Finally, 56 articles were verified to be eligible for our meta-analysis [16,17,18,19,20,21,22,23,24,25,26,27,28,29,30,31,32,33,34,35,36,37,38,39,40,41,42,43,44,45,46,47,48,49,50,51,52,53,54,55,56,57,58,59,60,61,62,63,64,65,66,67,68,69,70,71].

### 3.2. Characteristics of Qualified Studies

The basic information of the included articles is summarized in Table 1. The qualified studies were published between November 2001 and January 2024. Among these studies, 55 were case control studies conducted in 11 countries and regions: 10 in China [22,26,37,40,53,54,57,66,69,70], 13 in Korea [20,25,30,39,47,58,59,60,62,63,64,67,71], 18 in Japan [18,19,23,24,27,31,32,33,34,38,41,42,46,48,49,51,52,56], 3 in Poland [55,61,65], 2 in the U.S [36,50], 3 in England [16,17,45], 2 in Australia [21,35] (one involving ethnicities of Caucasian and Asian with the other only Caucasian) and 1 each in four other countries or regions [28,29,43,68]. These studies involved 10,804 cases with NTG and 217,540 controls in all. Data from one GWAS were available whose cohort consisted of 305 Japanese NTG patients and 355 healthy controls [44]. The NOS scores of all the studies were above 6 stars (thus qualifying for the meta-analysis). Genotype frequency and minor allele frequency are shown in Table S1. 

### 3.3. Meta-Analysis Results

Among all the SNPs extracted from the candidate gene literature, only 33 in 14 genetic loci were reported by at least two studies and met the criteria of this study. The association analysis and heterogeneity test in different genetic models are shown in Table 2 (since minor allele was opposite for SNP c.*1222C>T of *EDNRA* in the two studies incorporated, further analysis was not carried out in view of the heterogeneity. The related information is exhibited in Table S1). Of the 33 SNPs, 16 SNPs exhibited significant association with NTG, in which 11 variations (rs166850 of *OPA1*; rs10451941 of *OPA1*; rs735860 of *ELOVL5*; rs678350 of *HK2*; c.603T>A/Met98Lys of *OPTN*; c.412G>A/Thr34Thr of *OPTN*; rs10759930 of *TLR4*; rs1927914 of *TLR4*; rs1927911 of *TLR4*; c.*70C>G of *EDNRA* and rs1042522/-Arg72Pro of *P53*) showed positive NTG risk, whereas 5 others (rs2033008 of *NCK2*; rs3213787 of *SRBD1*; c.231G>A of *EDNRA*; rs10483727 of *SIX1-SIX6* and rs33912345 of *SIX1-SIX6*) showed negative correlation with the onset of NTG. 

#### 3.3.1. Gene Polymorphisms Associated with NTG

The source articles and sample size for analysis of each SNP were summarized in Table 2. 

##### *EDNRA* Polymorphisms

SNP c.-231G>A was associated with a decreased risk of NTG in the homozygote model (OR 0.61, 95%CI: 0.39–0.97, *p* = 0.035), but not in other models (Figure S1A).

SNP c.*70C>G was significantly associated with NTG in the dominant model (OR 1.67, 95%CI: 1.08–2.56, *p* = 0.020), but not in other models (Figure S1B).

##### *ELOVL5* Polymorphism

A significant association between rs735860 of *ELOVL5* gene and NTG was found in the heterozygote model (OR 1.51, 95%CI: 1.11–2.05, *p* = 0.009) (Figure S2A), but not in the other models (Figure S2B).

##### *HK2* Polymorphism

A significant association between rs678350 and NTG could be seen in all genetic models (allele: OR 1.54, 95%CI: 1.23–1.91, *p* < 0.001; dominant: OR 1.75, 95%CI: 1.32–2.31, *p* < 0.001; recessive: OR 1.75, 95%CI: 1.09–2.80, *p* = 0.020; heterozygote: OR 1.65, 95%CI: 1.22–2.23, *p* = 0.001 and homozygote: OR 2.14, 95%CI: 1.31–3.48, *p* = 0.002) (Figure S3).

##### *NCK2* Polymorphism

A significant association between rs2033008 and NTG could be seen in the allele (OR 0.70, 95%CI: 0.57–0.87, *p* = 0.001), recessive (OR 0.44, 95%CI: 0.27–0.70, *p* = 0.001) and homozygote models (OR 0.41, 95%CI: 0.25–0.67, *p* < 0.001) (Figure S4).

##### *OPA1* Polymorphisms

A significant association between rs166850 and NTG was found in three genetic models (allele: OR 1.49, 95%CI: 1.03–2.15, *p* = 0.034; dominant: OR 1.93, 95%CI: 1.09–3.45, *p* = 0.025 and heterozygote: OR 1.82, 95%CI: 1.04–3.19, *p* = 0.038) (Figure S5A), but no evidence of an association was found in other models (Figure S5C).

A significant association between rs10451941 and NTG was found in all genetic models (allele: OR 1.49, 95%CI: 1.30–1.71, *p* < 0.001; dominant: OR 1.55, 95%CI: 1.29–1.87, *p* < 0.001; recessive: OR 1.87, 95%CI: 1.43–2.45, *p* < 0.001; heterozygote: OR 1.41, 95%CI: 1.16–1.71, *p* = 0.001 and homozygote: OR 2.16, 95%CI: 1.59–2.95, *p* < 0.001) (Figure S5B).

##### *OPTN* Polymorphisms

For SNP c.603T>A/Met98Lys, random effects showed a significant association between it and NTG in the allele, dominant and heterozygote models (allele: OR 1.51, 95%CI: 1.14–2.02, *p* = 0.005; dominant: OR 1.55, 95%CI: 1.12–2.14, *p* = 0.007; heterozygote: OR 1.49, 95%CI: 1.07–2.07, *p* = 0.018), but no evidence of association was found in other models (Figure S6A).

Referring to SNP c.412G>A/Thr34Thr, a significant association was found in all genetic models (allele: OR 1.66, 95%CI: 1.29–2.13, *p* < 0.001; dominant: OR 1.69, 95%CI: 1.27–2.25, *p* < 0.001; recessive: OR 3.72, 95%CI: 1.41–9.79, *p* = 0.008; heterozygote: OR 1.58, 95%CI: 1.17–2.12, *p* = 0.002 and homozygote: OR 4.22, 95%CI: 1.59–11.18, *p* = 0.004) (Figure S6B).

The other three SNPs (IVS6-5T>C, IVS6-10G>A, IVS7+24G>A) exhibited no statistical significance with NTG (Figure S6C–G).

##### *P53* Polymorphism

A significant correlation of rs1042522/-Arg72Pro with NTG risk was revealed in the dominant model (OR 2.32, 95%CI: 1.02–5.28, *p* = 0.045), but not in the other four models (Figure S7).

##### *SRBD1* Polymorphism

A negative correlation of rs3213787 and NTG risk could be seen in allele (OR 0.40, 95%CI: 0.30–0.52, *p* = 0.001), dominant (OR 0.38, 95%CI: 0.26–0.51, *p* = 0.001) and heterozygote (OR 0.41, 95%CI: 0.30–0.56, *p* = 0.002) models but not in other models (Figure S8).

##### *TLR4* Polymorphisms

For rs10759930, results showed a significant association between it and NTG in heterozygote (OR 1.27, 95%CI: 1.02–1.59, *p* = 0.031) and homozygote models (OR 1.43, 95%CI: 1.06–1.94, *p* = 0.001) (Figure S9A).

For rs1927914, there was a significant association between it and NTG risk in the homozygote model (OR 1.43, 95%CI: 1.06–1.94, *p* = 0.020) (Figure S9B).

For rs1927911, a significant association between it and NTG risk was found in the heterozygote model (OR 1.29, 95%CI: 1.04–1.61, *p* = 0.021) (Figure S9C).

Rs12377632, rs2149356, rs11536889, rs7037117, rs7045953 revealed no significant association with NTG (Figure S9). 

##### *SIX1–SIX6* Polymorphism

Significant associations between rs10483727 and rs33912345 with a decreased risk of NTG could be seen in all models except for the heterozygote model (Figure S10A,B).

#### 3.3.2. Gene Polymorphisms Not Associated with NTG

Among all the genetic polymorphisms analyzed, 17 SNPs in 7 genes were found not to be statistically significant with NTG (see Table 2).

#### 3.3.3. Stratified Analysis in Different Ethnicities

In the stratification analysis by ethnicity, four SNPs were further investigated, including *MTHFR* rs397507444, *OPA1* rs166850 and rs10451941 as well as *p53* rs1042522. These SNPs showed no significant association with NTG in Asians. However, *OPA1* rs166850, *OPA1* rs10451941 and *p53* rs1042522 were significantly associated with NTG in Caucasians (Table S2).

### 3.4. Measurement of Publication Biases and Sensitivity Analysis

Begg’s Test did not reveal publication bias among the overall analysis for candidate SNPs and corresponding genes (*z* < 1.96, *p* > 0.05, Table 2), which strengthened the credibility of our results. In the sensitivity analysis, Suh’s study [47] was excluded for rs7037117 in the *TLR4* gene; this followed with a different conclusion that this SNP was significantly associated with NTG risk in the allele model (OR 1.46, 95%CI: 1.19–1.81, *p* < 0.001; *I*^2^ = 0.0%; Figure S11). Other alterations were not detected. 

## 4. Discussion

**Figure 2:**
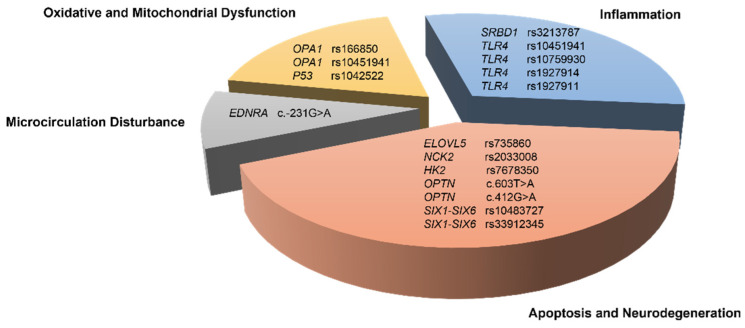
SNPs significantly associated with the risk of NTG and their possible biological functions.

Results showed that 16 SNPs in 10 genes were significantly associated with NTG in at least one genetic model. Related functions and pathogenic mechanisms of these associated alleles are summarized in Table 3 and Figure 2. 

### 4.1. Oxidative Stress-Related Genes

The *OPA1* gene encodes a kind of protein located in the inner membrane of mitochondria and plays an important role in cellular metabolism and activities, including stabilizing the mitochondrial construction, regulating mitochondrial fusion and fissure, taking part in oxidative phosphorylation and inhibiting chromosome c oxidase leaking, thus preventing cell apoptosis [72,73,74,75]. Aung [16] first conducted a study in Britain demonstrating that SNP rs166850 was significantly associated with NTG in 2002. We incorporated nine studies in our analysis with Caucasian, Asian and African-Caribbean populations, and finally elucidated that mutations in rs166850 and rs10451941 took effect in NTG in overall populations. This discovery reached the same conclusion as Guo’s meta-analysis in 2012 [76]. Compared with Guo, two more new studies were searched by us, thus confirming the reliability of the conclusion with a larger sample size. The interactions of the two polymorphisms with other genes may be a possible mechanism for NTG risk [65]. Interestingly, some scientists also found that TC/TC or CT/TT rs166850/rs10451941 combined genotype were more common in the Caucasian NTG population [16,45,65], which possibly indicated the overlapping pathogenetic effect of the two SNPs.

The *P53* gene lies on the chromosome 17p13.1, encoding transcription factor p53 which regulates the cell circle, cell metabolism and senescence as well as DNA repair [77,78,79]. It is also related to cell apoptosis by stimulating the transcriptional activity of redox-related genes and producing reactive oxygen species (ROS) which damage the physiological function of mitochondria [80]. SNP rs1042522 has been reported to be located in the proline-rich region of *p53* which would induce cell apoptosis by initiating the release of cytochrome c in the mitochondria into the cytosol [81]. Controversy exists about whether G allele or the mutant C allele would increase the susceptibility of POAG, with only different conclusions drawn in different ethnicities. 

### 4.2. Neurodegeneration and Apoptosis-Related Genes

*ELOVL5* is a member of the *ELOVL* gene family encoding a kind of elongase in the production of long-chain fatty acids [82], especially the polyunsaturated omega-3 and omega-6 fatty acids. The polyunsaturated fatty acids’ (PUFAs) metabolites play an important part in neurogenesis, neuronal survival and synaptic transmission [83,84,85]. What is more, ω-3 PUFAs could inhibit the damage of ischemia, inflammation, light, oxygen and age to retina [86]. Others showed that lack of eicosapentaenoic, docosahexaenoic acid and total ω-3 PUFAs were correlated to POAG risk [87]. The evidence above implies that alteration of rs735860 in the *ELOVL5* gene may increase NTG susceptibility by affecting the neurons’ metabolism and inducing apoptosis of retinal ganglion cells (RGCs). Overexpression of *ELOVL5* was also seen in prostate and gastric cancer cells for its incapability to regulate redox and mitochondrial homeostasis, and maintain appropriate production of reactive oxygen species (ROS) [88,89], which pointed out a new possible pathogenetic mechanism to be studied further. 

*NCK2* encodes proteins that regulate the cellular actin dynamics and polarity by interacting with tyrosine-phosphorylated growth factor receptors [90,91]. *NCK2* is demonstrated to exist in the ganglion cell layer, inner nuclear layer and outer plexiform layer, which are highest in the ganglion cell layer [51]. D2S176, which is located in the locus *GLC1B* and is only 24 kb from the gene *NCK2*, was found to be associated with a genetic heterogeneity of adult-onset POAG, and recently was considered to increase NTG risk in the Japanese population [92,93], which indicated the possible correlation of *NCK2* and NTG. In our study, the A allele in rs2033008 was negatively related to NTG onset in Korean and Japanese populations; we speculate that this variation changed the interaction of *NCK2* with other genes resulting in a defensive effect of RGCs. Shi et al. [51] found that this SNP was associated with NTG but not POAG and supposed that the mechanisms of NTG were focused on optic nerve damage, but for POAG, changes in the anterior chamber weighed more heavily.

The *HK2* gene is located in the outer membrane of mitochondria and catalyzes the first step of glycolysis [51]. It is expressed widely in photoreceptors (PRs) and plays a role in the aerobic glycolysis metabolizing glucose entering the cells [94]. *HK2* inhibits the release of cytochrome c to prevent apoptosis through the Bax/Bak pathway [95]. Zhou et al. [96] found that the decreased expression of *HK2* would lead to irreversible rod degeneration in animal models. Given the importance of *HK2*-encoding proteins in mitochondria, it is reasonable to believe that the variant phenotypes could induce metabolic dysfunction and, furthermore, optic neuropathy. 

*OPTN* is a 67 kDa protein which is expressed in many cells and tissues, especially in retina, brain, heart and skeleton muscle [97]. It acts as an adaptor protein and participates in many physiological activities such as signal transduction, cell division, cell survival, exocytosis, autophagy, protein trafficking and so on [97]. Mutations of *OPTN* have been widely considered a pathogenesis of POAG [98] as well as NTG [99,100], of which E50K (c.148G>A) is the most common to be associated with POAG, and another mutation H486R (c.1457A>G) is correlated with juvenile open-angle glaucoma (JOAG). In our study, we drew a conclusion that c.603T>A and c.412G>A in OPTN were significantly associated with NTG, but another POAG meta-analysis [101] only found the association between the former with NTG in the stratified analysis. The reason may lie in the difference in studies included: the POAG meta-analysis included four studies, while we included three studies for one of the four failed to define NTG clearly and was thus excluded.

*SIX1-SIX6* belong to the *SIX* gene family containing two protein domains, which could encode homeobox domain transcription factors and may play a role in regulating the development of the visual system [102]. Studies have shown that a missense variant in rs33912345 of *SIX6* was associated with RNFL thinning [103,104], suggesting its function in RGC development or degeneration. The possible mechanism lies in its interaction with CDNK2A/CDNK2B and subsequently triggering RGC loss [105,106]. Our results, finding that the risk allele mutations of both rs10483727 and rs33912345 were associated with NTG, were consistent with the findings of previous studies [104,107], which confirmed the results of this research.

### 4.3. Inflammation-Related Genes

*SRBD1* encodes proteins which modulate signal transduction via binding with RNA. Its overexpression is considered to promote proinflammatory cytokines accumulation, prevent cell proliferation and accelerate cell apoptosis [108,109,110], which would do harm to RGCs in NTG. Kanemaki et al. stated that *SRBD1* polymorphisms were associated with NTG, despite IOP [111], suggesting the different pathogenetic factors of NTG from hyper-tension glaucoma (HTG). Rs3213787 was revealed to be negatively correlated with NTG, which indicates that the G allele may reduce *SRBD1* activity and protect RGC from apoptosis.

Toll-like receptors (TLRs) are a kind of pattern recognition receptor (PRR) which play an important role in innate immunity and initiate inflammatory response by recognizing and binding with pathogen-associated molecular patterns (PAMPs) [112]. Among them, TLR4 is expressed in the conjunctiva, cornea, uvea and retina [49]. A study found the overexpression of TLR4 in glaucomatous retina and the optic nerve [113], which indicates that inflammation and chronic stress would have an effect on the microenvironment of RGCs, change the construction of lamina cribrosa and increase the susceptibility of remaining axons, leading to irreversible optic neuropathy. Recently, it was suggested that *TLR4* was associated with POAG for its activation generates meshwork fibrosis via the TGF-β pathway, leading to elevation of IOP [114]; in addition, ligands of TLR4 (e.g., LPS and HSP) were considered as candidate antigens of NTG [115]. In our study, rs10759930, rs1927914 and rs1927911 were seen to show a significant association with NTG; we speculate that these polymorphisms change the expression of some important proteins by altering the translated regions or intron regions of mRNA in the translation process.

### 4.4. Microcirculation Disturbance-Related Gene

EDNRA is the specific receptor of endothelial-1 (ET-1), a 21-amino acid peptide performing as a vasoconstrictor [116], and can mediate ET-1 level in retinal blood flow. ET systems express greatly in most ocular tissues [117,118]. There have been studies which reported higher ET-1 concentration in the plasma of NTG patients compared with that of controls [119,120]. ET-1 system activation causes vasospasm, vascular endothelial injury and microvascular lesion, thus damaging the optic nerve. In addition, ET-1 affects the morphology and physiology of the optic nerve in rabbit models, resulting in optic disc excavation, loss of axons and demyelination of the optic nerve despite the level of IOP [121,122]. It also inhibits the anterograde axonal transport, lowers neural metabolic activity and promotes astrocytes’ proliferation, which is responsible for the optic neuropathy in glaucoma [123]. 

Concerns regarding the limitation of utilizing duplicated datasets from the same researchers or groups (ex. Study 2, 8, 10 and 11 shown in Table 1) were also taken into account. In some specific scenarios, these overlapping data should be selected for further utilization according to standard, otherwise bias may occur if the same subject is incorporated repeatedly. In view of this, we searched further similar literature for advice [101,124,125]. As a result, we found that those SNP-associated meta-analyses also incorporated studies from the “same dataset”. It seems reasonable because the overlapping data were not really included in the analysis for a specific SNP. Though duplicated in the cohort information in some studies, they were independent from each other because they targeted different genes and SNPs. Hence, a great deal of information would be missed once these data were deleted.

In this study, we summarized the reported genotype polymorphisms and obtained an insight into SNPs’ association with the susceptibility to NTG. We adopted some measures such as Quality assessment, HWE test, Begg’s Test and sensitivity analysis to control possible statistical errors and assure the credibility of our meta-analysis. However, there are some limitations which should not be ignored in the meta-analysis. First, the sample size from different ethnicities should be enlarged. Second, only studies published in English met the inclusion criteria, which might cause a failure to incorporate other non-English articles, resulting in incomplete analysis. Finally, the functions and mechanisms of specific allele variants were not clearly explained, partly due to the different results of included articles and limited experimental evidence. Further studies should be conducted to explain the doubts.

## 5. Conclusions

In conclusion, the present study summarized the reported genotype polymorphisms and obtained an insight into SNPs’ association with susceptibility to NTG. The mechanisms of these mutations on NTG could possibly be attributed to changing the metabolisms and activities of RGCs via mitochondria functional alteration, inflammation and immunity. Experimental evidence and more large-scale studies are required for a greater understanding of these genes and polymorphisms.

## Figures and Tables

**Table 1:** Characteristics of qualified studies involved in the meta-analysis.

No.	Reference	Country/City (Ethnicity)	Sample Size		Male/Female		Age, y		Quality Assessment	Genotyping Methods	Genotype Frequency					
											Cases			Controls		
			Cases	Controls	Cases	Controls	Cases	Controls			AA	AB	BB	AA	AB	BB
1	Lee et al., 2022 [70]	China Taiwan (Chinese)	222	236	122/100	127/109	69 ± 9	68 ± 10	8☆	allelic	126	80	16	108	101	27
2	Shin et al., 2022 [71]	Korea (Korean)	210	117	NA	NA	NA	NA	8☆	allelic	130	76	4	64	45	8
3	He et al., 2022 [69]	China Hongkong (Chinese)	537	496	278/259	184/312	63.2 ± 12.8	70.2 ± 10.8	9☆	allelic	NA	NA	NA	NA	NA	NA
		China Shantou (Chinese)	135	543	79/56	283/260	61.6 ± 14.6 7	74.4 ± 6.9								
4	Liuska et al., 2021 [68]	Finland (Finnish)	892	205,435	NA	NA	NA	NA	9☆	allelic	884	8	0	204,378	1053	4
5	Kim et al., 2021 [67]	Korea (Korean)	282	213	127/155	120/93	54.3 ± 13.3	54.6 ± 9.7	9☆	allelic	NA	NA	NA	NA	NA	NA
6	Milanowski et al., 2021 [65]	Poland (Caucasian)	204	258	48/156	80/178	71.6 ± 11.1	70.9 ± 11.6	8☆	allelic	121	70	0	168	79	6
7	Yue et al., 2021 [66]	China (Chinese)	402	425	226/176	254/171	63.8 ± 6.5	64.5 ± 5.1	7☆	allelic	311	79	12	338	79	8
8	Jung et al., 2020 [63]	Korea (Korean)	159	103	60/99	44/59	61.14 ± 11.94	68.78 ± 9.82	7☆	allelic	260	44	1	241	96	18
9	Lee et al., 2020 [64]	Korea (Korean)	435	419	206/229	231/188	58.8 ± 13.6	56.2 ± 10.3	7☆	allelic	288	127	20	290	116	13
10	Jung et al., 2019 [60]	Korea (Korean)	154	101	58/96	42/59	61.23 ± 11.95	67.29 ±11.37	7☆	allelic	70	68	16	62	31	8
11	Jung et al., 2019 [62]	Korea (Korean)	157	106	57/100	43/63	61.06 ± 12.16	67.19 ± 10.53	7☆	allelic	148	9	0	98	8	0
12	Kosior-Jarecka et al., 2019 [61]	Poland (Caucasian)	143	165	43/100	NA	74	NA	8☆	allelic	77	57	6	90	68	6
13	Jeoung et al., 2017 [58]	Korea (Korean)	245	231	117/128	115/116	60.2 ± 12.7	58.6 ± 12.4	8☆	allelic	211	39	1	212	33	0
14	Suh et al., 2017 [59]	Korea (Korean)	140	352	NA	NA	NA	NA	7☆	allelic	62	61	16	158	151	33
15	Nishisako et al., 2016 [56]	Japan (Japanese)	292	500	140/152	246/254	46.7 ± 8.4	50.2 ± 10.6	7☆	allelic	93	135	64	147	248	105
16	Gao et al., 2016 [54]	China (Chinese)	55	50	29/26	31/19	52.5 ± 14.0	49.1 ± 13.6	7☆	allelic	39	15	1	38	12	0
17	Sang et al., 2016 [57]	China (Chinese)	181	266	104/77	114/152	53.5 ± 16.8	67.6 ± 11.3	7☆	allelic	131	45	5	140	103	23
18	Kosior-Jarecka et al., 2016 [55]	Poland (Caucasian)	160	165	50/110	50/115	72.01 ± 11.61	72.52 ± 11.06	6☆	allelic	83	6	71	81	15	69
19	Lin et al., 2014 [53]	China (Chinese)	249	262	123/117	140/122	63.2 ± 10.2	61.3 ± 11.4	6☆	allelic	231	18	0	241	21	0
20	Shi et al., 2013 [52]	Japan (Japanese)	163	180	86/77	95/85	61.8 ± 13.7	68.0 ± 7.7	6☆	allelic	147	16	0	168	11	1
21	Shi et al., 2013 [51]	Japan (Japanese)	stage 1 120	121	61/60	61/59	54.0 ± 12.2	70.3 ± 10.2	6☆	allelic	159	111	16	130	105	36
			stage 2 286	271	139/147	145/126	56.4 ± 13.3	69.7 ± 9.3		allelic						
22	Wiggs et al., 2012 [50]	U.S. (Caucasian)	64	400	23/41	179/221	61.06 ± 11.6	66.06 ± 11.3	7☆	allelic	36	15	1	82	72	13
23	TAKANO et al., 2012 [49]	Japan (Japanese)	365	216	171/194	116/100	58.6 ± 13.1	69.7 ± 11.3	8☆	allelic	141	159	65	103	85	28
24	Suh et al., 2011 [47]	Korea (Korean)	147	380	NA	NA	NA	NA	9☆	allelic	52	72	23	126	191	63
25	Mabuchi et al., 2011 [46]	Japan (Japanese)	158	191	65/93	70/121	68.6 ± 11.8	65.7 ± 11.4	7☆	allelic	51	84	23	71	89	31
26	Yasumura et al., 2011 [48]	Japan (Japanese)	295	518	142/153	NA	46.4 ± 8.1	NA	8☆	allelic	241	52	2	404	110	4
27	Wolf et al., 2010 [43]	Germany (German)	273	280	96/177	115/165	63.9 ± 14.2	66 ± 13	7☆	allelic	74	131	68	75	135	10
28	Meguro et al., 2010 [44]	Japan (Japanese)	305	355	145/160	174/181	46.6 ± 8.5	61.7 ± 8.9	8☆	genomic	51	138	116	100	162	93
29	Mabuchi et al., 2010 [42]	Japan (Japanese)	213	191	91/122	70/121	NA	NA	7☆	allelic	79	100	34	77	84	30
30	Mabuchi et al., 2010 [41]	Japan (Japanese)	213	191	91/122	70/121	NA	NA	7☆	allelic	59	107	47	49	84	58
31	Fan et al., 2010 [40]	China (Chinese)	100	201	54/46	120/81	63.2 ± 11.5	69.8 ± 8.7	8☆	allelic	89	9	1	173	27	1
32	Yu-Wai-Man et al., 2010 [45]	England (Caucasian)	70	75	NA	NA	NA	79.3	7☆	allelic	41	26	3	59	13	3
33	Fan et al., 2009 [37]	China (Chinese)	42	77	33/9	58/19	66.7 ± 10.1	72.0 ± 8.5	8☆	allelic	27	13	2	47	27	3
34	Clement et al., 2009 [35]	Australia (75 Caucasian, 1 Asian)	34	42	9/25	16/26	72.5 ± 9.4	70.4 ± 7.8	9☆	allelic	21	11	2	25	14	3
35	Daugherty et al., 2009 [36]	U.S. (Caucasian)	52	167	18/34	62/105	69.8 ± 12.0	60.3 ± 12.0	7☆	allelic	29	28	5	109	57	12
36	Mabuchi et al., 2009 [38]	Japan (Japanese)	213	189	91/122	70/119	63.9 ± 13.7	65.5 ± 11.4	7☆	allelic	92	95	26	83	83	23
37	Woo et al., 2009 [39]	Korea (Korean)	78	100	32/46	47/53	46.2 ± 11.7	49.3 ± 9.2	8☆	allelic	25	34	19	31	50	19
38	Shibuya et al., 2008 [34]	Japan (Japanese)	250	318	119/131	157/161	46.1 ± 7.7	61.2 ± 8.3	8☆	allelic	81	127	42	137	141	40
39	Tosaka et al., 2007 [33]	Japan (Japanese)	290	241	142/148	114/127	55.8 ± 13.0	69.7 ± 11.3	7☆	allelic	106	130	54	67	130	44
40	Mabuchi et al., 2007 [31]	Japan (Japanese)	194	185	NA	NA	63.6 ± 13.3	65.3 ± 11.5	8☆	allelic	190	4	0	182	3	0
41	Miyazawa et al., 2007 [32]	Japan (Japanese)	103	118	53/50	62/56	61.8 ± 11.7	68.0 ± 7.7	7☆	allelic	76	25	2	72	41	5
42	Jeoung et al., 2007 [30]	Korea (Korean)	67	100	28/39	47/53	48.8 ± 10.2	49.3 ± 9.2	8☆	allelic	53	13	1	83	16	1
43	How et al., 2007 [29]	Singapore (Chinese)	94	79	64/30	32/47	72.9	67.7	7☆	allelic	71	17	1	64	13	2
44	Kim et al., 2006 [25]	Korea (Korean)	67	100	28/39	47/53	48.8 ± 10.2	49.3 ± 9.2	8☆	allelic	29	32	6	44	39	17
45	Lam et al., 2006 [26]	China (Chinese)	106	300	NA	191/109	NA	70.4 ± 9.3	7☆	allelic	102	3	1	286	13	1
46	Inagaki et al., 2006 [24]	Japan (Japanese)	294	240	144/150	114/126	58.8 ± 13.2	69.7 ± 11.2	7☆	allelic	219	72	3	176	63	1
47	Mabuchi et al., 2006 [27]	Japan (Japanese)	131	106	NA	NA	62.8 ± 13.3	65.0 ± 10.5	7☆	allelic	54	58	19	48	39	19
48	Yao et al., 2006 [28]	Africa (African-Caribbean)	61	48	NA	NA	52.1	61.3	9☆	allelic	58	3	0	46	2	0
49	Hashizume et al., 2005 [23]	Japan (Japanese)	268	240	129/139	113/127	58.8 ± 13.4	69.7 ± 11.2	7☆	allelic	164	90	14	163	66	11
50	Dimasi et al., 2005 [21]	Australia (Caucasian)	62	178	NA	NA	NA	NA	8☆	allelic	34	43	22	38	108	55
51	Fan et al., 2005 [22]	China (Chinese)	106	281	NA	180/101	NA	69.8 ± 9.8	6☆	allelic	67	36	3	200	74	7
52	Funayama et al., 2004 [18]	Japan (Japanese)	217	218	97/120	92/126	60.3 ± 12.4	70.6 ± 10.9	7☆	allelic	169	43	5	182	35	1
53	Fuse et al., 2004 [19]	Japan (Japanese)	65	100	27/38	62/38	61.8 ± 13.7	68 ± 7.7	6☆	allelic	55	9	1	95	5	0
54	Woo et al., 2004 [20]	Korea (Korean)	65	101	26/39	48/53	47.0 ± 10.3	49.0 ± 9.2	8☆	allelic	62	3	0	101	0	0
55	Powell et al., 2003 [17]	England (Caucasian)	61	168	26/35	109/59	NA	NA	6☆	allelic	41	16	4	111	53	4
56	Aung et al., 2002 [16]	England (Caucasian)	163	186	NA	NA	NA	NA	7☆	allelic	57	26	0	86	14	0

NA: not applicable. ☆: The quality of studies was recorded in the form of stars and the maximum star was 9.

**Table 2:** Significant association analysis of genetic polymorphisms with NTG.

No.	Gene Symbol	SNP	Minor Allele	No. of Cohorts	Ethnicity	Pooled Sample Size		Genetic Model	Heterogeneity Test		Fixed or Random Effect Model	OR	95%CI	*p*	Begg’s Test	
						Cases	Controls		*p* (Q)	*I*² *(*%)					*z*	*p*
1	*APOE*	-491A>T	T	2	Chinese	312	581	B vs. A	0.928	0.0	fixed	0.91	0.44–1.88	0.800	0.000	1.000
								BB + AB vs. AA	0.933	0.0	fixed	0.77	0.35–1.73	0.531	0.000	1.000
								BB vs. AA + AB	0.974	0.0	fixed	2.76	0.39–19.68	0.312	0.000	1.000
								AB vs. AA	0.940	0.0	fixed	0.63	0.25–1.54	0.307	0.000	1.000
								BB vs. AA	0.973	0.0	fixed	2.71	0.38–19.36	0.321	0.000	1.000
		-427T>C	C	2		312	582	B vs. A	0.941	0.0	fixed	0.50	0.11–2.25	0.365	0.000	1.000
								BB + AB vs. AA	0.940	0.0	fixed	0.50	0.11–2.25	0.363	0.000	1.000
								BB vs. AA + AB	excluded	excluded	NA	NA	NA	NA	NA	NA
								AB vs. AA	0.940	0.0	fixed	0.50	0.11–2.25	0.363	0.000	1.000
								BB vs. AA	excluded	excluded	NA	NA	NA	NA	NA	NA
		-219T>G	G	2		312	581	B vs. A	0.885	0.0	fixed	0.98	0.78–1.25	0.899	0.000	1.000
								BB + AB vs. AA	0.865	0.0	fixed	0.80	0.59–1.10	0.172	0.000	1.000
								BB vs. AA + AB	0.957	0.0	fixed	1.65	1.01–2.71	0.046	0.000	1.000
								AB vs. AA	0.874	0.0	fixed	0.70	0.50–0.98	0.039	0.000	1.000
								BB vs. AA	0.916	0.0	fixed	1.39	0.83–2.33	0.215	0.000	1.000
2	*EDNRA*	c.-231G>A	A	2	Caucasian Korean	227	265	B vs. A	0.578	0.0	fixed	0.95	0.73–1.22	0.683	0.000	1.000
								BB + AB vs. AA	0.731	0.0	fixed	0.86	0.60–1.22	0.384	0.000	1.000
								BB vs. AA + AB	0.128	56.7	random	0.95	0.64–1.42	0.816	0.000	1.000
								AB vs. AA	0.057	72.4	random	0.85	0.50–1.45	0.554	0.000	1.000
								BB vs. AA	0.118	59.0	random	**0.61**	**0.39–0.97**	**0.035**	0.000	1.000
		c.*70C>G	G	2		227	265	B vs. A	0.005	87.4	random	1.29	0.60–2.78	0.513	0.000	1.000
								BB + AB vs. AA	0.168	47.4	fixed	**1.67**	**1.08–2.56**	**0.020**	0.000	1.000
								BB vs. AA + AB	0.008	86.0	random	1.14	0.37–3.56	0.817	0.000	1.000
								AB vs. AA	0.769	0.0	fixed	1.45	0.89–2.35	0.135	0.000	1.000
								BB vs. AA	0.026	79.7	random	1.44	0.44–4.72	0.543	0.000	1.000
3	*ELOVL5*	rs735860	C	2	Japanese	463	546	B vs. A	0.001	91.3	random	1.49	0.79–2.80	0.216	0.000	1.000
								BB + AB vs. AA	0.015	83.2	random	1.81	0.88–3.70	0.106	0.000	1.000
								BB vs. AA + AB	0.049	74.2	random	1.29	0.67–2.49	0.447	0.000	1.000
								AB vs. AA	0.448	0.0	fixed	**1.51**	**1.11–2.05**	**0.009**	0.000	1.000
								BB vs. AA	0.030	78.7	random	1.65	0.71–3.82	0.246	0.000	1.000
4	*HK2*	rs678350	G	2	Korean Japanese	440	372	B vs. A	0.580	0.0	fixed	**1.54**	**1.23–1.91**	**0.000**	0.000	1.000
								BB + AB vs. AA	0.689	0.0	fixed	**1.75**	**1.32–2.31**	**0.000**	0.000	1.000
								BB vs. AA + AB	0.499	0.0	fixed	**1.75**	**1.09–2.80**	**0.020**	0.000	1.000
								AB vs. AA	0.482	0.0	fixed	**1.65**	**1.22–2.23**	**0.001**	0.000	1.000
								BB vs. AA	0.633	0.0	fixed	**2.14**	**1.31–3.48**	**0.002**	0.000	1.000
5	*NCK2*	rs2033008	A	2	Korean Japanese	440	372	B vs. A	0.799	0.0	fixed	**0.70**	**0.57–0.87**	**0.001**	0.000	1.000
								BB + AB vs. AA	0.636	0.0	fixed	0.77	0.58–1.02	0.065	0.000	1.000
								BB vs. AA + AB	0.526	0.0	fixed	**0.44**	**0.27–0.70**	**0.001**	0.000	1.000
								AB vs. AA	0.968	0.0	fixed	0.86	0.64–1.16	0.321	0.000	1.000
								BB vs. AA	0.558	0.0	fixed	**0.41**	**0.25–0.67**	**0.000**	0.000	1.000
6	*MTHFR*	rs397507444, c.677 C/T	T	3	Caucasian Asian Korean Japanese	243	248	B vs. A	0.923	0.0	fixed	1.02	0.79–1.33	0.855	0.000	1.000
								BB + AB vs. AA	0.828	0.0	fixed	1.05	0.73–1.52	0.778	0.000	1.000
								BB vs. AA + AB	0.517	0.0	fixed	1.00	0.62–1.62	0.986	0.000	1.000
								AB vs. AA	0.582	0.0	fixed	1.07	0.72–1.60	0.725	0.000	1.000
								BB vs. AA	0.813	0.0	fixed	1.01	0.59–1.72	0.969	0.000	1.000
		rs1217691063c.1298 A/C	C	2	Korean Japanese	209	206	B vs. A	0.604	0.0	fixed	0.94	0.65–1.34	0.720	0.000	1.000
								BB + AB vs. AA	0.572	0.0	fixed	0.95	0.63–1.43	0.797	0.000	1.000
								BB vs. AA + AB	0.712	0.0	fixed	0.55	0.12–2.65	0.459	0.000	1.000
								AB vs. AA	0.574	0.0	fixed	0.97	0.64–1.47	0.873	0.000	1.000
								BB vs. AA	0.511	0.0	fixed	0.63	0.13–2.97	0.556	0.000	1.000
7	*NOS3*	rs1799983, 894 G>T	T	2	Korean Chinese	350	446	B vs. A	0.248	25.0	fixed	1.03	0.71–1.47	0.888	0.000	1.000
								BB + AB vs. AA	0.219	33.7	fixed	1.00	0.68–1.46	0.989	0.000	1.000
								BB vs. AA + AB	0.865	0.0	fixed	2.43	0.30–19.58	0.404	0.000	1.000
								AB vs. AA	0.215	35.0	fixed	0.97	0.66–1.43	0.879	0.000	1.000
								BB vs. AA	0.833	0.0	fixed	2.38	0.30–19.03	0.414	0.000	1.000
		rs2070744, -786T>C	C	2		350	446	B vs. A	0.315	0.8	fixed	1.04	0.75–1.43	0.816	0.000	1.000
								BB + AB vs. AA	0.363	0.0	fixed	1.00	0.70–1.42	0.987	0.000	1.000
								BB vs. AA + AB	0.261	20.7	fixed	1.97	0.52–7.39	0.315	0.000	1.000
								AB vs. AA	0.427	0.0	fixed	0.96	0.67–1.37	0.814	0.000	1.000
								BB vs. AA	0.248	25.1	fixed	1.92	0.51–7.17	0.334	0.000	1.000
8	*OPA1*	rs166850, IVS8+4C⬎T	T	9	Caucasian Chinese Japanese Korean African-Caribbean	904	1217	B vs. A	0.034	52.1	random	**1.49**	**1.03–2.15**	**0.034**	0.940	0.348
								BB + AB vs. AA	0.000	76.1	random	**1.93**	**1.09–3.45**	**0.025**	0.520	0.602
								BB vs. AA + AB	0.174	39.6	fixed	0.96	0.41–2.24	0.931	1.020	0.308
								AB vs. AA	0.000	73.0	random	**1.82**	**1.04–3.19**	**0.038**	0.310	0.754
								BB vs. AA	0.216	32.6	fixed	1.04	0.44–2.43	0.930	1.020	0.308
		rs10451941, IVS8+32T⬎C	C	9		944	1220	B vs. A	0.405	3.5	fixed	**1.49**	**1.30–1.71**	**0.000**	0.100	0.917
								BB + AB vs. AA	0.243	22.5	fixed	**1.55**	**1.29–1.87**	**0.000**	1.150	0.251
								BB vs. AA + AB	0.603	0.0	fixed	**1.87**	**1.43–2.45**	**0.000**	0.300	0.764
								AB vs. AA	0.130	36.0	fixed	**1.41**	**1.16–1.71**	**0.001**	0.520	0.602
								BB vs. AA	0.564	0.0	fixed	**2.16**	**1.59–2.95**	**0.000**	0.000	1.000
9	*OPTN*	c.603T>A, Met98Lys	A	3	Chinese Japanese	388	599	B vs. A	0.239	30.1	fixed	**1.51**	**1.14–2.02**	**0.005**	1.040	0.296
								BB + AB vs. AA	0.341	7.0	fixed	**1.55**	**1.12–2.14**	**0.007**	1.040	0.296
								BB vs. AA + AB	0.417	0.0	fixed	2.20	0.82–5.95	0.119	1.040	0.296
								AB vs. AA	0.401	0.0	fixed	**1.49**	**1.07–2.07**	**0.018**	0.000	1.000
								BB vs. AA	0.459	0.0	fixed	2.41	0.88–6.58	0.087	0.000	1.000
		c.412G>A, Thr34Thr	A	3		388	599	B vs. A	0.185	40.8	fixed	**1.66**	**1.29–2.13**	**0.000**	0.000	1.000
								BB + AB vs. AA	0.346	5.7	fixed	**1.69**	**1.27–2.25**	**0.000**	0.000	1.000
								BB vs. AA + AB	0.272	23.3	fixed	**3.72**	**1.41–9.79**	**0.008**	0.000	1.000
								AB vs. AA	0.529	0.0	fixed	**1.58**	**1.17–2.12**	**0.002**	0.000	1.000
								BB vs. AA	0.258	26.3	fixed	**4.22**	**1.59–11.18**	**0.004**	0.000	1.000
		IVS6-5T>C	C	2		171	381	B vs. A	0.048	74.4	random	1.26	0.64–2.50	0.507	0.000	1.000
								BB + AB vs. AA	0.141	53.8	random	1.07	0.59–1.97	0.817	0.000	1.000
								BB vs. AA + AB	0.135	55.3	random	2.08	0.53–8.18	0.296	0.000	1.000
								AB vs. AA	0.058	72.2	random	2.03	0.98–4.17	0.055	0.000	1.000
								BB vs. AA	0.387	0.0	fixed	1.01	0.41–2.50	0.976	0.000	1.000
		IVS6-10G>A	A	2		171	381	B vs. A	0.532	0.0	fixed	1.31	0.79–2.18	0.296	0.000	1.000
								BB + AB vs. AA	0.499	0.0	fixed	1.33	0.78–2.27	0.299	0.000	1.000
								BB vs. AA + AB	.	.	fixed	1.55	0.10–25.17	0.759	NA	NA
								AB vs. AA	0.474	0.0	fixed	1.32	0.77–2.28	0.316	0.000	1.000
								BB vs. AA	.	.	fixed	1.56	0.10–25.40	0.757	NA	NA
		IVS7+24G>A	A	2		171	381	B vs. A	0.760	0.0	fixed	1.58	0.99–2.51	0.053	0.000	1.000
								BB + AB vs. AA	0.148	52.3	random	1.29	0.60–2.75	0.517	0.000	1.000
								BB vs. AA + AB	0.398	0.0	fixed	2.75	0.51–14.87	0.241	0.000	1.000
								AB vs. AA	0.061	71.6	random	1.17	0.42–3.32	0.761	0.000	1.000
								BB vs. AA	0.856	0.0	fixed	2.71	0.49–14.92	0.253	0.000	1.000
10	*p53*	rs1042522, -Arg72Pro	C	5	Caucasian Chinese Japanese	490	1135	B vs. A	0.000	81.3	random	0.97	0.64–1.45	0.868	0.240	0.806
								BB + AB vs. AA	0.000	93.3	random	**2.32**	**1.02–5.28**	**0.045**	0.240	0.806
								BB vs. AA + AB	0.013	68.5	random	1.140	0.58–2.25	0.704	0.240	0.806
								AB vs. AA	0.003	75.1	random	0.880	0.53–1.46	0.630	-0.240	1.000
								BB vs. AA	0.001	79.3	random	1.020	0.41–2.51	0.973	-0.240	1.000
11	*SRBD1*	rs3213787	G	3	Korean Japanese	622	649	B vs. A	0.050	66.6	random	**0.40**	**0.30–0.52**	**0.001**	0.000	1.000
								BB + AB vs. AA	0.080	60.4	random	**0.38**	**0.26–0.51**	**0.001**	0.000	1.000
								BB vs. AA + AB	0.082	60.1	random	0.23	0.09–0.59	0.252	0.000	1.000
								AB vs. AA	0.067	63.0	random	**0.41**	**0.30–0.56**	**0.002**	0.000	1.000
								BB vs. AA	0.075	61.4	random	0.20	0.08–0.50	0.201	0.000	1.000
12	*TLR4*	rs10759930	C	3	Korean Japanese	762	914	B vs. A	0.087	59.0	random	1.21	0.97–1.53	0.097	1.040	0.296
								BB + AB vs. AA	0.095	57.6	random	1.29	0.94–1.78	0.114	0.000	1.000
								BB vs. AA + AB	0.186	40.5	fixed	1.20	0.91–1.58	0.192	0.000	1.000
								AB vs. AA	0.178	42.1	fixed	**1.27**	**1.02–1.59**	**0.031**	0.000	1.000
								BB vs. AA	0.151	47.1	fixed	**1.43**	**1.06–1.94**	**0.001**	1.040	0.296
		rs1927914	G	3		762	914	B vs. A	0.002	84.2	random	1.29	0.89–1.87	0.180	1.040	0.296
								BB + AB vs. AA	0.074	61.7	random	1.32	0.94–1.85	0.104	0.000	1.000
								BB vs. AA + AB	0.489	0.0	fixed	1.24	0.94–1.64	0.125	1.040	0.296
								AB vs. AA	0.129	51.1	random	1.30	0.94–1.78	0.108	1.040	0.296
								BB vs. AA	0.172	43.1	fixed	**1.43**	**1.06–1.94**	**0.020**	0.000	1.000
		rs1927911	A	3		762	914	B vs. A	0.001	85.5	random	1.33	0.91–1.96	0.141	0.000	1.000
								BB + AB vs. AA	0.091	58.2	random	1.31	0.95–1.80	0.102	1.040	0.296
								BB vs. AA + AB	0.486	0.0	fixed	1.25	0.94–1.66	0.125	0.000	1.000
								AB vs. AA	0.155	46.3	fixed	**1.29**	**1.04–1.61**	**0.021**	0.000	1.000
								BB vs. AA	0.013	76.9	random	1.48	0.78–2.79	0.227	1.040	0.296
		rs12377632	T	3		762	914	B vs. A	0.114	54.0	random	1.16	0.93–1.44	0.181	1.040	0.296
								BB + AB vs. AA	0.060	64.4	random	1.27	0.90–1.80	0.171	0.000	1.000
								BB vs. AA + AB	0.379	0.0	random	1.08	0.81–1.45	0.589	0.000	1.000
								AB vs. AA	0.057	65.0	random	1.29	0.89–1.87	0.179	0.000	1.000
								BB vs. AA	0.016	75.8	random	1.46	0.79–2.72	0.231	0.000	1.000
		rs2149356	T	3		762	914	B vs. A	0.020	74.5	random	1.25	0.93–1.68	0.133	0.000	1.000
								BB + AB vs. AA	0.057	65.0	random	1.29	0.91–1.83	0.155	1.040	0.296
								BB vs. AA + AB	0.359	2.3	fixed	1.31	0.99–1.74	0.062	0.000	1.000
								AB vs. AA	0.120	52.8	random	1.24	0.90–1.71	0.181	0.000	1.000
								BB vs. AA	0.019	74.9	random	1.47	0.80–2.72	0.219	1.040	0.296
		rs11536889	C	3		762	914	B vs. A	0.315	13.5	fixed	1.05	0.89–1.24	0.527	0.000	1.000
								BB + AB vs. AA	0.000	93.8	random	0.86	0.37–1.98	0.720	1.040	0.296
								BB vs. AA + AB	0.508	0.0	fixed	0.88	0.57–1.35	0.559	1.040	0.296
								AB vs. AA	0.448	0.0	fixed	1.13	0.92–1.40	0.247	0.000	1.000
								BB vs. AA	0.017	75.5	random	1.34	0.59–3.02	0.867	0.000	1.000
		rs7037117	G	3		762	914	B vs. A	0.000	89.1	random	1.12	0.67–1.89	0.665	1.040	0.296
								BB + AB vs. AA	0.051	66.4	random	1.34	0.93–1.92	0.112	1.040	0.296
								BB vs. AA + AB	0.746	0.0	fixed	1.28	0.82–2.01	0.280	0.000	1.000
								AB vs. AA	0.055	65.6	random	1.33	0.91–1.93	0.138	1.040	0.296
								BB vs. AA	0.079	60.6	random	1.48	0.71–3.08	0.290	1.040	0.296
		rs7045953	G	3		762	914	B vs. A	0.307	15.4	fixed	1.12	0.86–1.45	0.414	1.040	0.296
								BB + AB vs. AA	0.339	7.5	fixed	1.11	0.84–1.47	0.467	1.040	0.296
								BB vs. AA + AB	0.506	0.0	fixed	1.56	0.51–4.75	0.436	0.000	1.000
								AB vs. AA	0.393	0.0	fixed	1.09	0.82–1.46	0.534	1.040	0.296
								BB vs. AA	0.491	0.0	fixed	1.42	0.46–4.35	0.542	0.000	1.000
13	*WDR36*	rs17553936, IVS16–30A>G	G	2	Chinese Japanese	145	195	B vs. A	0.287	11.6	fixed	0.70	0.47–1.04	0.078	0.000	1.000
								BB + AB vs. AA	0.365	0.0	fixed	0.65	0.41–1.03	0.068	0.000	1.000
								BB vs. AA + AB	0.440	0.0	fixed	0.68	0.20–2.28	0.528	0.000	1.000
								AB vs. AA	0.469	0.0	fixed	0.66	0.41–1.06	0.086	0.000	1.000
								BB vs. AA	0.378	0.0	fixed	0.61	0.18–2.06	0.426	0.000	1.000
14	*SIX1-SIX6*	rs10483727	C	2	Korean Japanese	391	383	B vs. A	0.141	53.8	random	**0.55**	**0.38–0.80**	**0.002**	0.000	1.000
								BB + AB vs. AA	0.062	71.2	random	**0.56**	**0.32–0.99**	**0.047**	0.000	1.000
								BB vs. AA + AB	0.543	0.0	fixed	**0.25**	**0.12–0.54**	**0.000**	0.000	1.000
								AB vs. AA	0.041	76.0	random	0.65	0.34–1.24	0.188	0.000	1.000
								BB vs. AA	0.748	0.0	fixed	**0.21**	**0.10–0.46**	**0.000**	0.000	1.000
		rs33912345	A	2		391	383	B vs. A	0.069	69.7	random	**0.56**	**0.35–0.89**	**0.013**	0.000	1.000
								BB + AB vs. AA	0.067	70.3	random	**0.55**	**0.32–0.77**	**0.038**	0.000	1.000
								BB vs. AA + AB	0.856	0.0	fixed	**0.24**	**0.11–0.54**	**0.001**	0.000	1.000
								AB vs. AA	0.086	66.1	random	0.62	0.36–1.08	0.089	0.000	1.000
								BB vs. AA	0.696	0.0	fixed	**0.20**	**0.08–0.45**	**0.000**	0.000	1.000

NTG: normal tension glaucoma; SNP: single nucleotide polymorphism; OR: odds ratio; CI: confidence interval; NA: not applicable. Bold value: OR (95%CI) >1 or <1 with *p* < 0.05.

**Table 3:** Possible functions and pathogenic mechanisms of the associated SNPs in the development of NTG.

Gene		SNP	OR (95%CI)	*p* Value	Involved Mechanisms	Possible Function in NTG
Name	Symbol					
optic atrophy 1	*OPA1*	rs166850, IVS8+4C⬎T	1.49 (1.03–2.15)	0.034	Encoding proteins crucial for normal mitochondrial function	Downregulation of OPA1 gene is associated with increased mitochondrial fission in optic nerve, increasing cell death of RGC-5 cells
		rs10451941, IVS8+32T⬎C	1.49 (1.30–1.71)	0.000		
elongation of long-chain fatty acids family member 5	*ELOVL5*	rs735860	1.51 (1.11–2.05)	0.009	Encoding elongases of polyunsaturated fatty acids	Enhanced ELOVL5 expression may cause apoptosis and cell growth inhibition in retinal ganglion cells
non-catalytic region of tyrosine kinase adaptor	*NCK2*	rs2033008	0.70 (0.57–0.87)	0.001	Regulating the cellular actin dynamics and polarity	Participating in neural regeneration and protection, especially for the transition of glia cells into photoreceptors
hexokinase 2	*HK2*	rs678350	1.54 (1.23–1.91)	0.000	Catalyzing the first step of glycolysis	Important for photoreceptors’ function and preventing cell apoptosis
optineurin	*OPTN*	c.603T>A, Met98Lys	1.51 (1.14–2.02)	0.005	An adaptor protein involved in many cellular functions	Inhibition of autophagy and induced cell death of RGCs
		c.412G>A, Thr34Thr	1.66 (1.29–2.13)	0.000		
S1 RNA binding domain 1	*SRBD1*	rs3213787	0.40 (0.30–0.52)	0.001	Modulating signal transduction	Prevent cell proliferation, promote proinflammatory cytokines accumulation and accelerate cell apoptosis of RGCs
toll-like receptor 4	*TLR4*	rs10759930	1.27 (1.02–1.59)	0.031	Participating in innate immunity and initiating inflammatory response	Inflammation and immunity lead to RGC apoptosis and optic nerve damage
		rs1927914	1.43 (1.06–1.94)	0.020		
		rs1927911	1.29 (1.04–1.61)	0.021		
endothelin receptor type A	*EDNRA*	c.-231G>A	0.61 (0.39–0.97)	0.035	Bind with ET-1 to activate vasoconstriction	Damaging optic nerve resulted from vascular dysfunction and promoting astrocytes proliferation
		c.*70C>G	1.67 (1.08–2.56)	0.020		
tumor protein p53	*p53*	rs1042522, -Arg72Pro	2.32 (1.02–5.28)	0.045	Regulating cell circle, cell metabolism, senescence and DNA repair	Producing ROS causing mitochondria damage and inducing cell apoptosis
sine oculis homeobox homolog 1- sine oculis homeobox homolog 6	*SIX1-SIX6*	rs10483727	0.55 (0.38–0.80)	0.002	Regulating the development of the visual system	Reducing the number of retinal ganglion cells, especially during the aging process
		rs33912345	0.56 (0.35–0.89)	0.013		

NTG: normal tension glaucoma; SNP: single nucleotide polymorphism; OR: odds ratio; CI: confidence interval; *OPA1*: optic atrophy 1; *ELOVL5*: elongation of long-chain fatty acids family member 5; *NCK2*: non-catalytic region of tyrosine kinase adaptor 2; *HK2*: hexokinase 2; *OPTN*: optineurin; *SRBD1*: S1 RNA binding domain 1; *TLR4*: toll-like receptor 4; *EDNRA*: endothelin receptor type A; *p53*: tumor protein 53.

## Data Availability

The authors confirm that the data supporting the findings of this study are available within the article and its Supplementary Materials.

## Supplementary Materials

The following supporting information can be downloaded at: https://www.mdpi.com/article/10.3390/genes15040491/s1, Figure S1. Associations between SNPs in EDNRA gene with NTG onset; Figure S2. Associations between SNPs in *ELOVL5* gene with NTG onset; Figure S3. Associations between SNPs in *HK2* gene with NTG onset; Figure S4. Associations between SNPs in *NCK2* gene with NTG onset; Figure S5. Associations between SNPs in *OPA1* gene with NTG onset; Figure S6. Associations between SNPs in *OPTN* gene with NTG onset; Figure S7. Associations between SNPs in *P53* gene with NTG onset; Figure S8. Associations between SNPs in *SRBD1* gene with NTG onset; Figure S9. Associations between SNPs in *TLR4* gene with NTG onset; Figure S10. Associations between SNPs in *SIX1-SIX6* gene with NTG onset; Figure S11. Sensitivity analysis for rs7037117 in *TLR4* gene; Table S1. Genotype frequencies for candidate SNPs in the involved studies; Table S2. Genetic associations of NTG in different ethnicities.
