# Supplementary material for: Association of Gene Polymorphisms with Normal Tension Glaucoma: A Systematic Review and Meta-Analysis
Source: Genes (Basel). 2024 Apr 14;15(4):491. doi: 10.3390/genes15040491 (PMC11050218; doi:10.3390/genes15040491)
Supplement: Supplementary file 1 [file genes-15-00491-s001.zip › Supplementary figures.pdf]

Figure S1. Associations between SNPs in *EDNRA* gene with NTG onset.

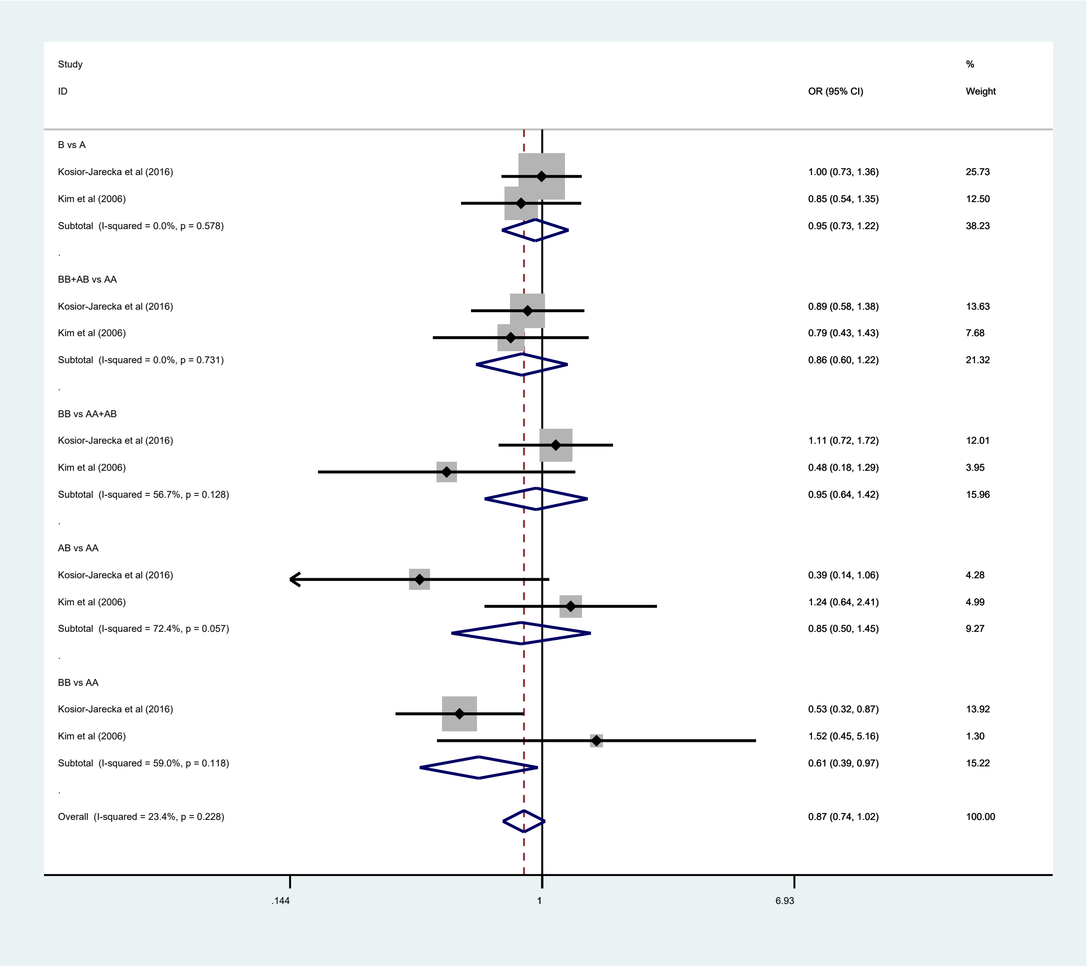

A) Forest plots exhibiting the association between *EDNRA* (c.-231G>A) and NTG in allele (B vs A), dominant (BB+AB vs AA), recessive (BB vs AA+AB) and co-dominant (AB vs AA & BB vs AA) models respectively.

OR: odds ratio; CI: confidence interval; NTG: normal tension glaucoma.

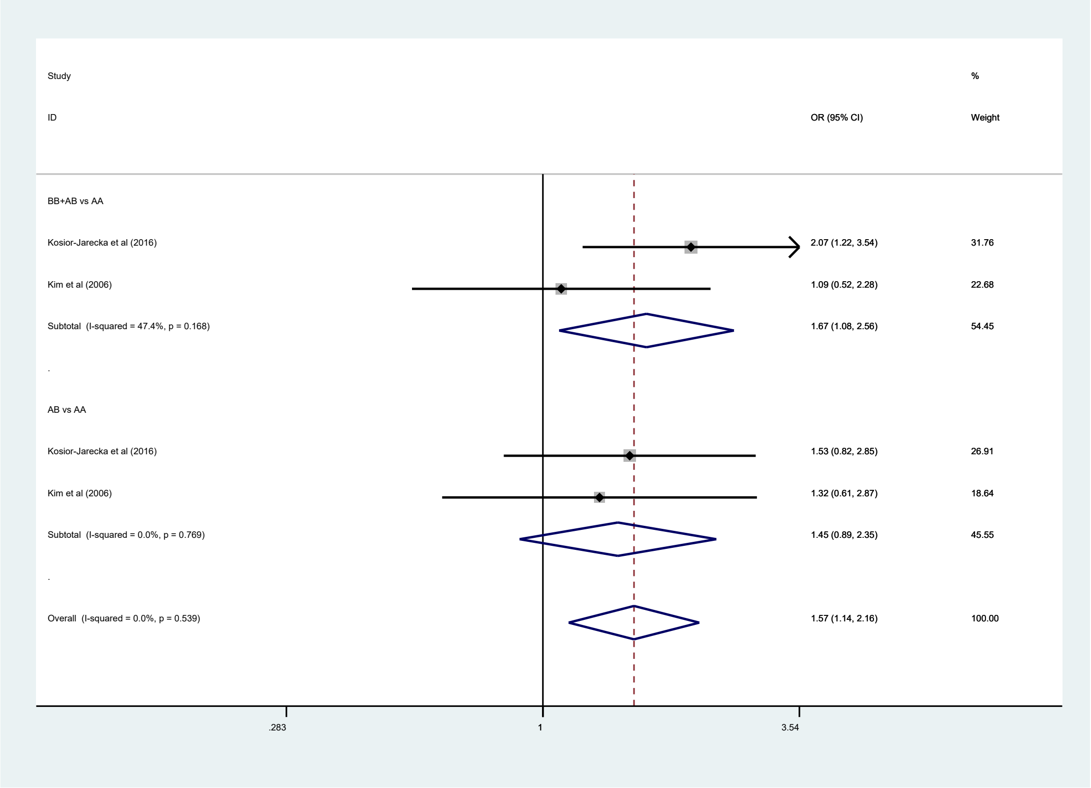

B) Forest plots exhibiting the association between *EDNRA* (c.*70C>G) and NTG in dominant (BB+AB vs AA) and co-dominant (AB vs AA) models respectively (from fixed effects analysis). OR: odds ratio; CI: confidence interval; NTG: normal tension glaucoma.

Figure S2. Associations between SNPs in *ELOVL5* gene with NTG onset.

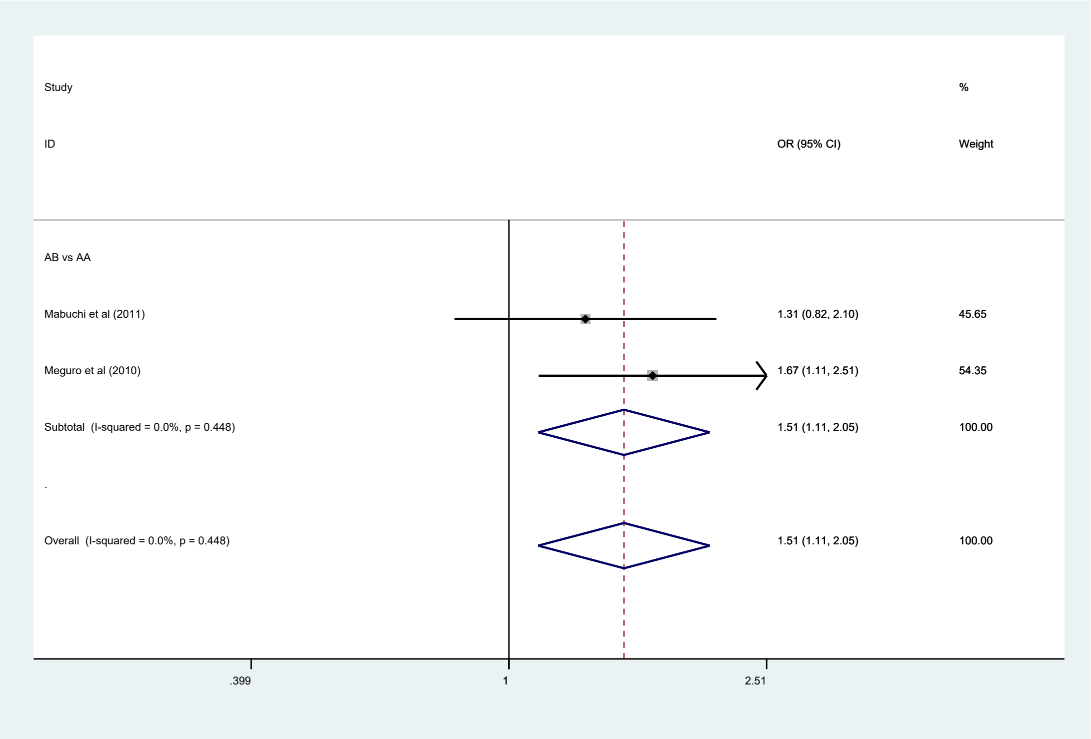

A) Forest plots demonstrating the association between *ELOVL5* (rs735860) and NTG in heterozygote model (AB vs AA).
OR: odds ratio; CI: confidence interval; NTG: normal tension glaucoma.

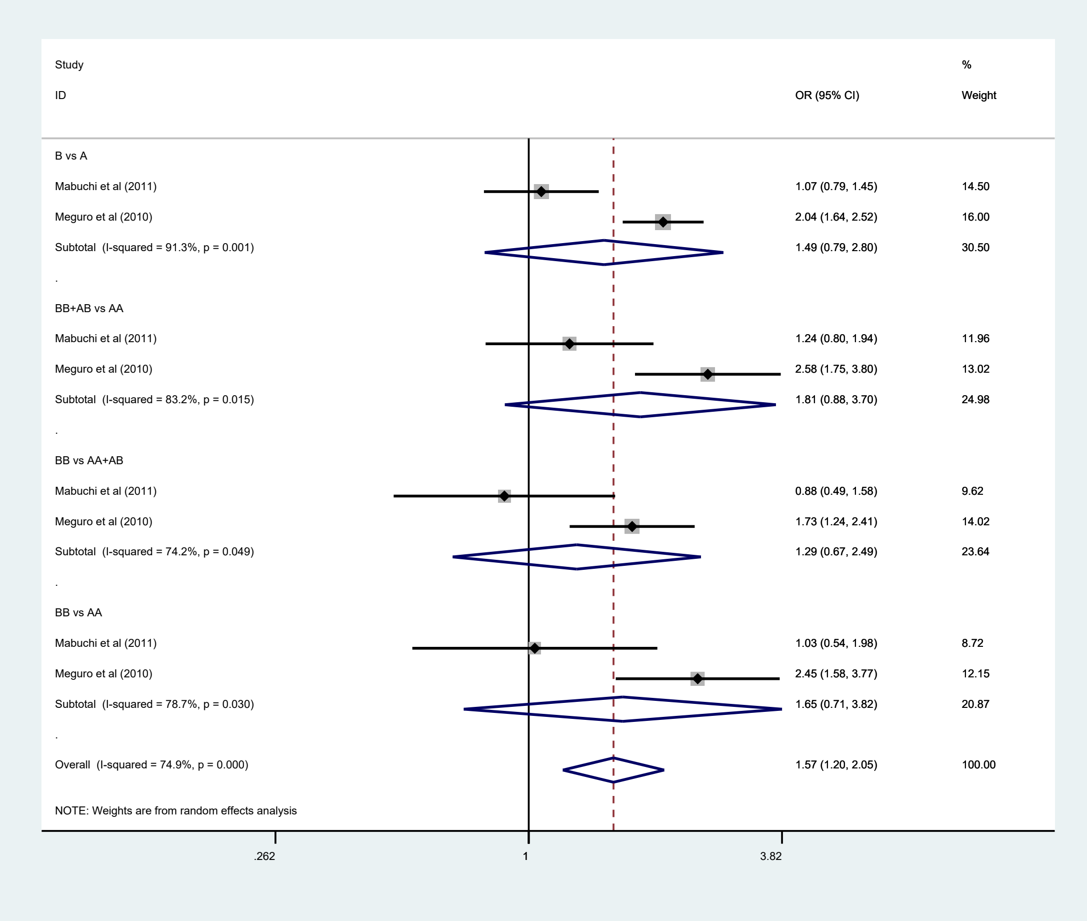

B) Forest plots demonstrating no association between *ELOVL5* (rs735860) and NTG in allele (B vs A), dominant (BB+AB vs AA), recessive (BB vs AA+AB) and homozygote (BB vs AA) models respectively (from random effects analysis).
OR: odds ratio; CI: confidence interval; NTG: normal tension glaucoma.

Figure S3. Associations between SNPs in *HK2* gene with NTG onset.

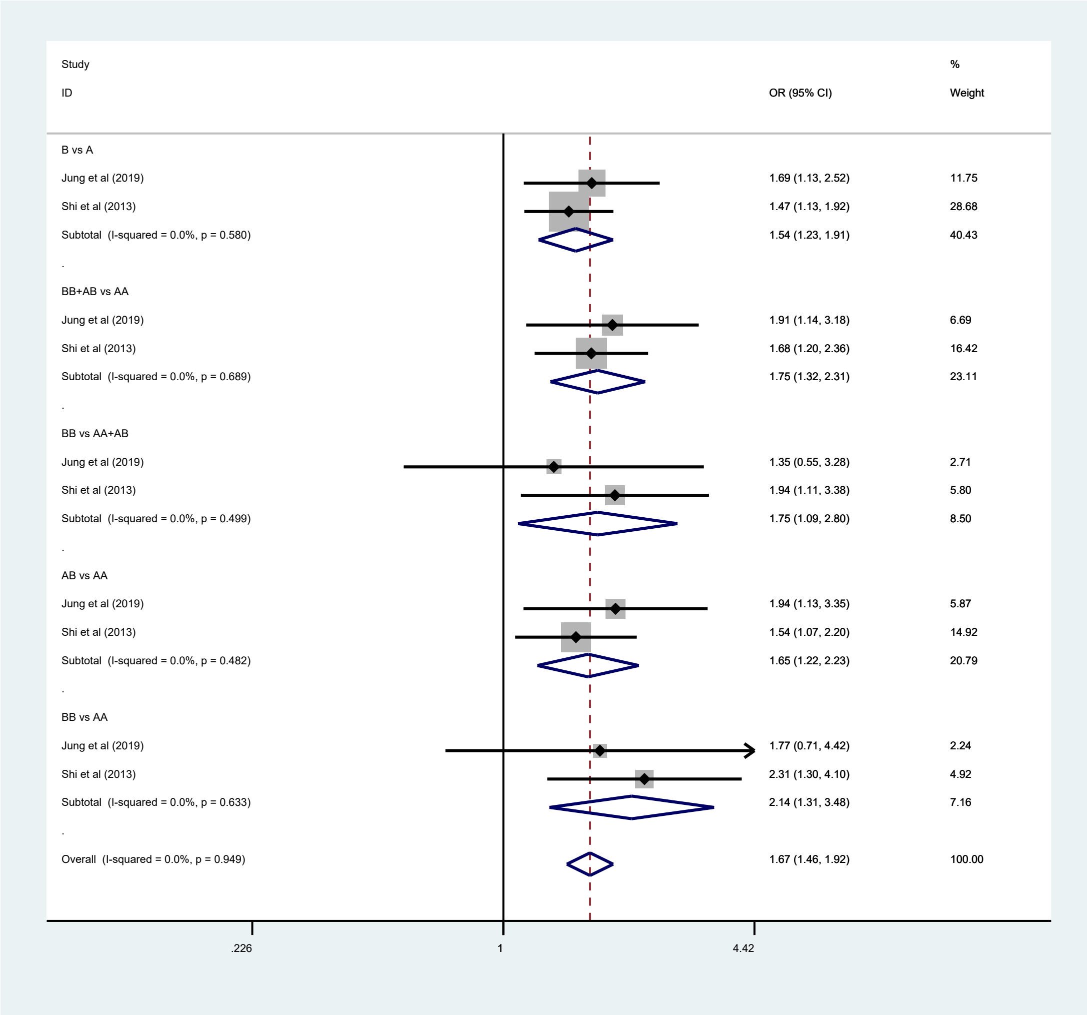

Forest plots demonstrating the association between *HK2* (rs678350) and NTG in allele (B vs A), dominant (BB+AB vs AA), recessive (BB vs AA+AB) and co-dominant (AB vs AA & BB vs AA) models respectively.

OR: odds ratio; CI: confidence interval; NTG: normal tension glaucoma.

Figure S4. Associations between SNPs in *NCK2* gene with NTG onset.

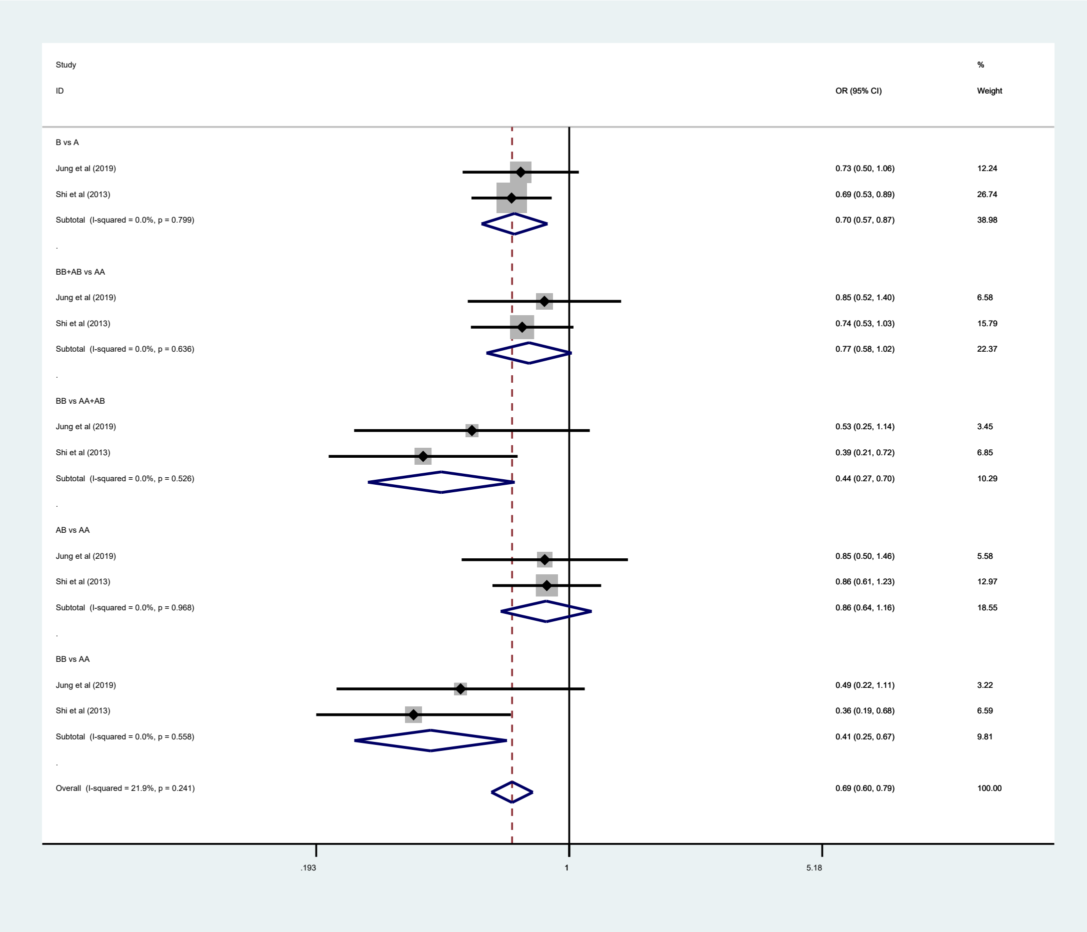

Forest plots demonstrating the association between *NCK2* (rs2033008) and NTG in allele (B vs A), dominant (BB+AB vs AA), recessive (BB vs AA+AB) and co-dominant (AB vs AA & BB vs AA) models respectively.

OR: odds ratio; CI: confidence interval; NTG: normal tension glaucoma.

Figure S5. Associations between SNPs in *OPA1* gene with NTG onset.

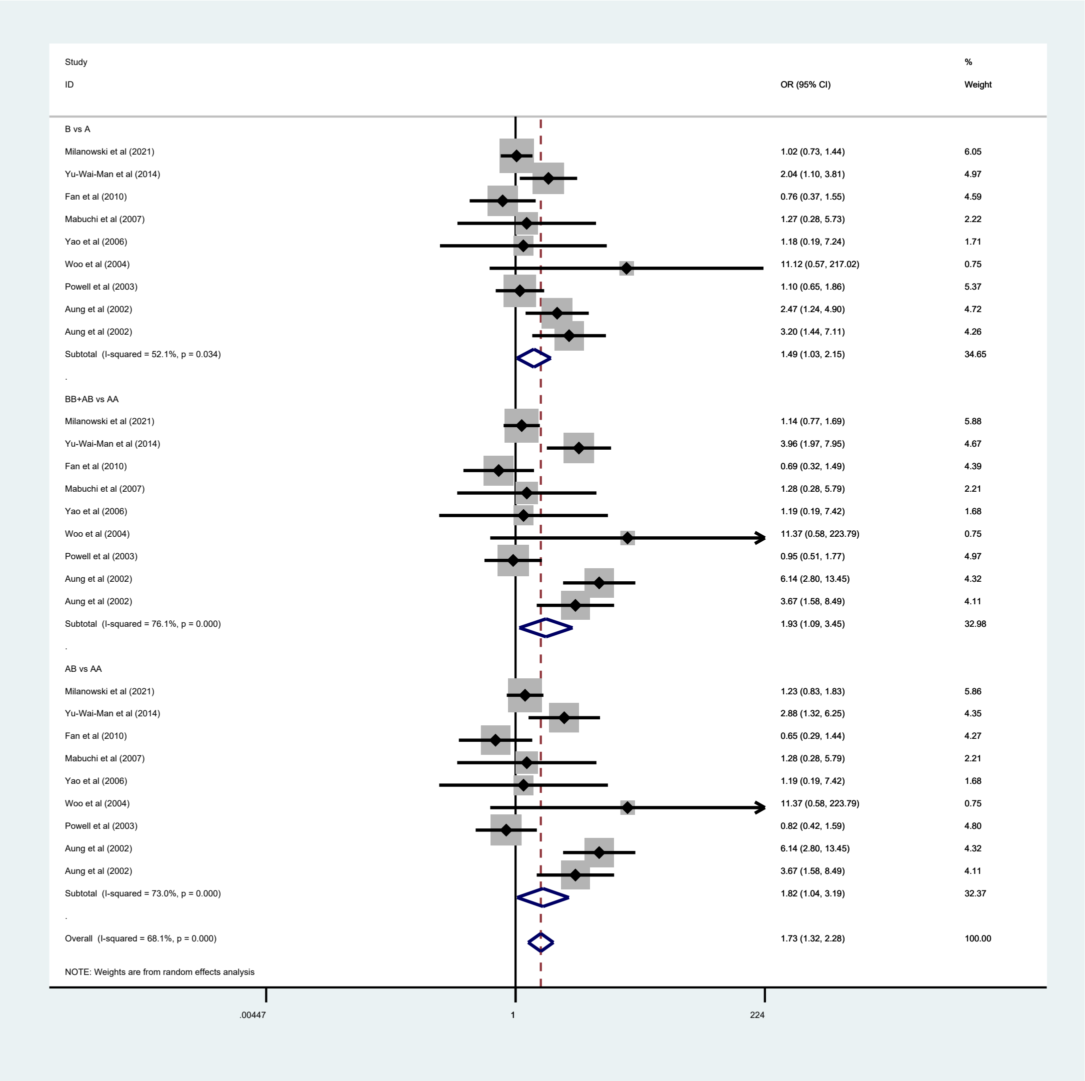

A) Forest plots demonstrating the association between *OPA1* (rs166850, IVS8+4C¬T) and NTG in allele (B vs A), dominant (BB+AB vs AA) and heterozygote (AB vs AA) models respectively (from random effects analysis).

OR: odds ratio; CI: confidence interval; NTG: normal tension glaucoma.

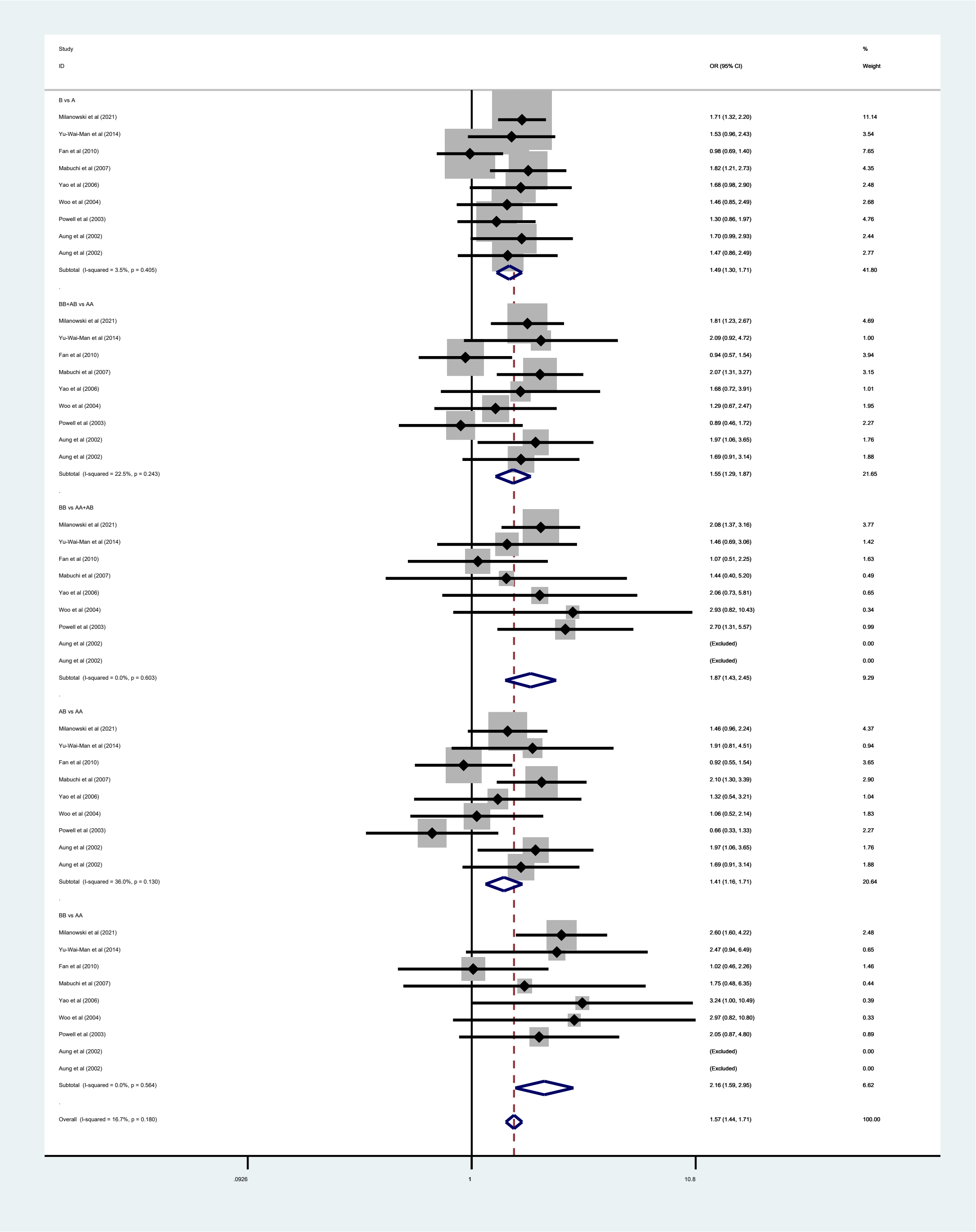

B) Forest plots demonstrating the association between *OPA1* (rs10451941, IVS8+32T¬C) and NTG in allele (B vs A), dominant (BB+AB vs AA), recessive (BB vs AA+AB) heterozygote (AB vs AA) and homozygote (BB vs AA) models respectively.

OR: odds ratio; CI: confidence interval; NTG: normal tension glaucoma.

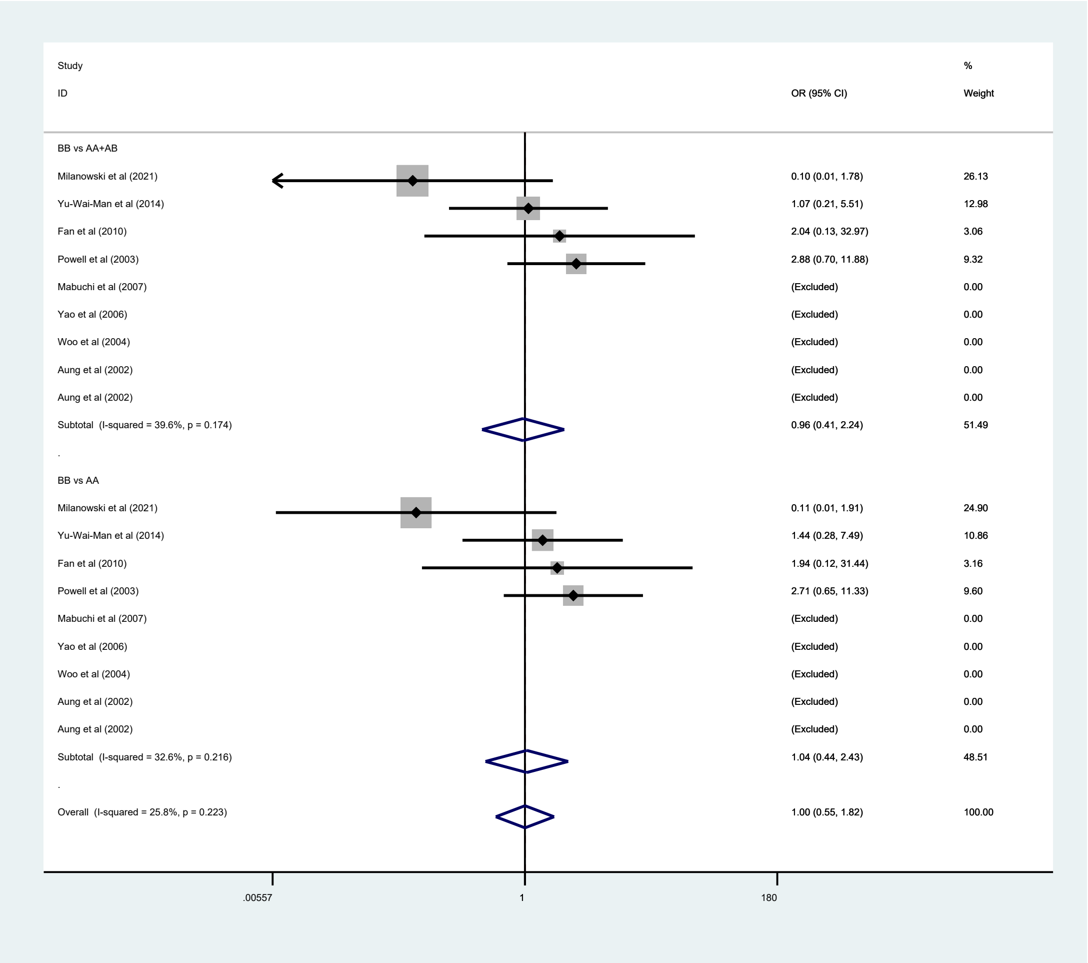

C) Forest plots demonstrating no association between *OPA1* (rs166850, IVS8+4C¬T) and NTG in recessive (BB vs AA+AB) and homozygote (BB vs AA) models respectively (from fixed effects analysis).

OR: odds ratio; CI: confidence interval; NTG: normal tension glaucoma.

Figure S6. Associations between SNPs in *OPTN* gene with NTG onset.

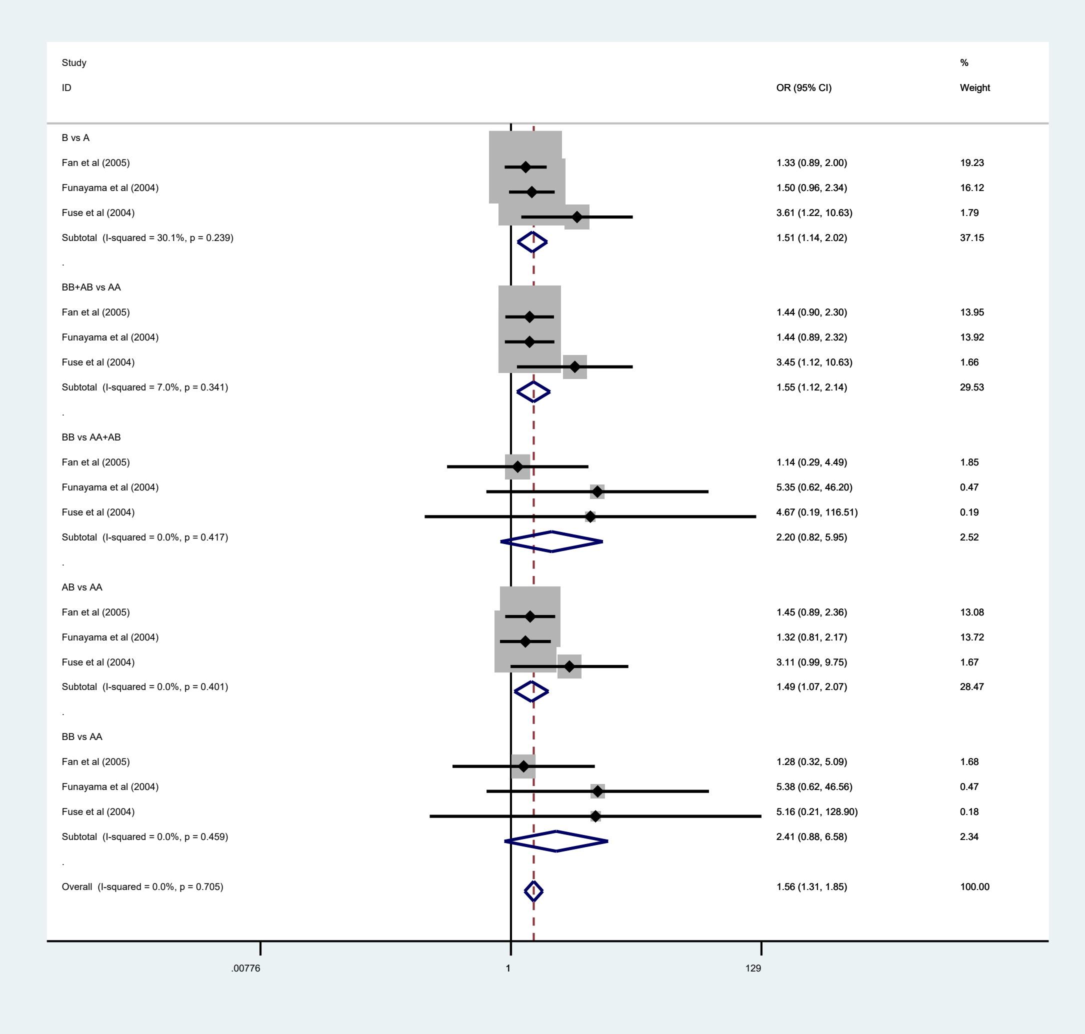

A) Forest plots demonstrating the association between *OPTN* (c.603T>A, Met98Lys) and NTG in allele (B vs A), dominant (BB+AB vs AA), recessive (BB vs AA+AB) and co-dominant (AB vs AA & BB vs AA) models respectively.

OR: odds ratio; CI: confidence interval; NTG: normal tension glaucoma.

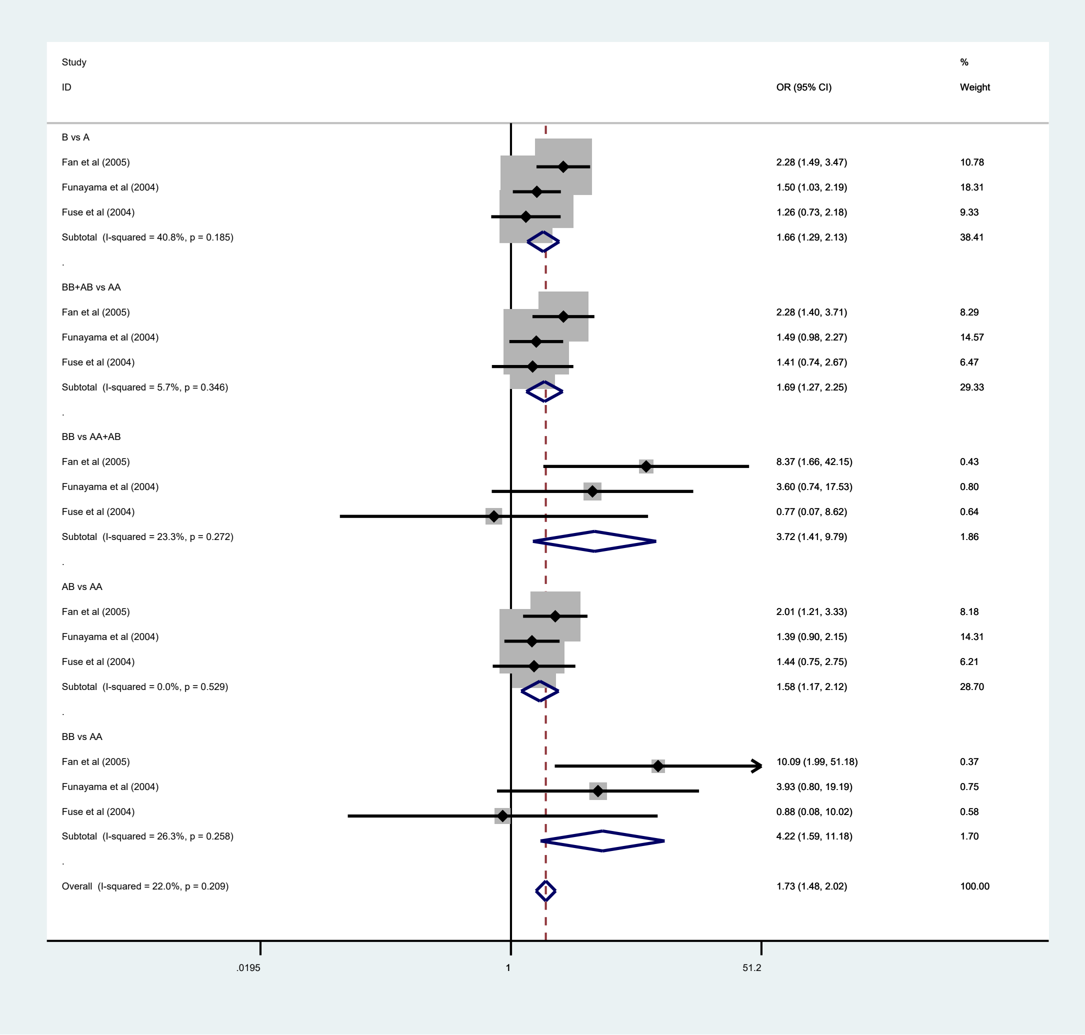

B) Forest plots demonstrating the association between *OPTN* (c.412G>A, Thr34Thr) and NTG in allele (B vs A), dominant (BB+AB vs AA), recessive (BB vs AA+AB) and co-dominant (AB vs AA & BB vs AA) models respectively.

OR: odds ratio; CI: confidence interval; NTG: normal tension glaucoma.

IVS6-5T>C

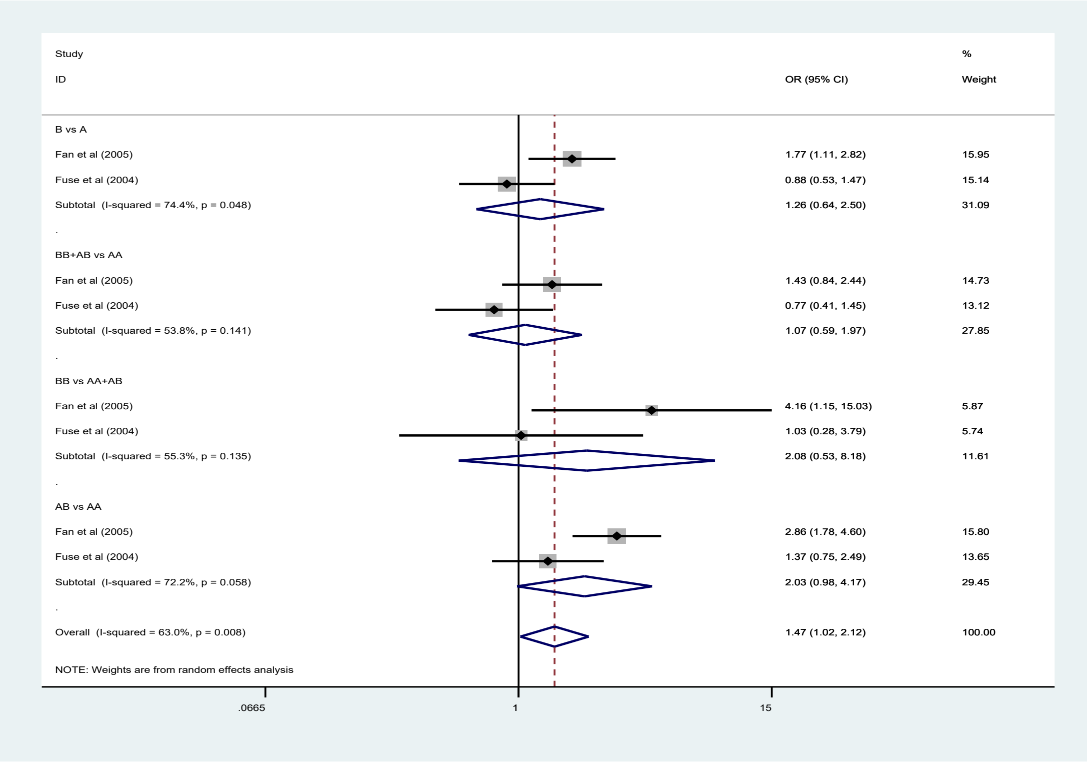

C) Forest plots demonstrating no association between *OPTN* (IVS6-5T>C) and NTG in allele (B vs A), dominant (BB+AB vs AA), recessive (BB vs AA+AB) or heterozygote (AB vs AA) models respectively (from random effects analysis).
OR: odds ratio; CI: confidence interval; NTG: normal tension glaucoma.

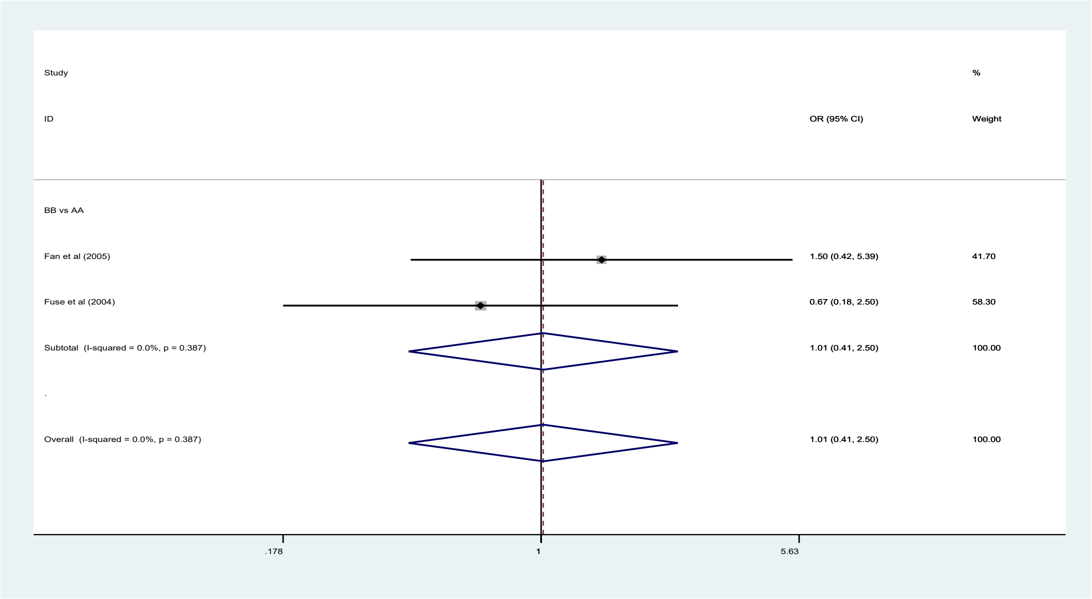

D) Forest plots demonstrating no association between *OPTN* (IVS6-5T>C) and NTG in homozygote (BB vs AA) model (from fixed effect analysis).
OR: odds ratio; CI: confidence interval; NTG: normal tension glaucoma.

IVS6-10G>A

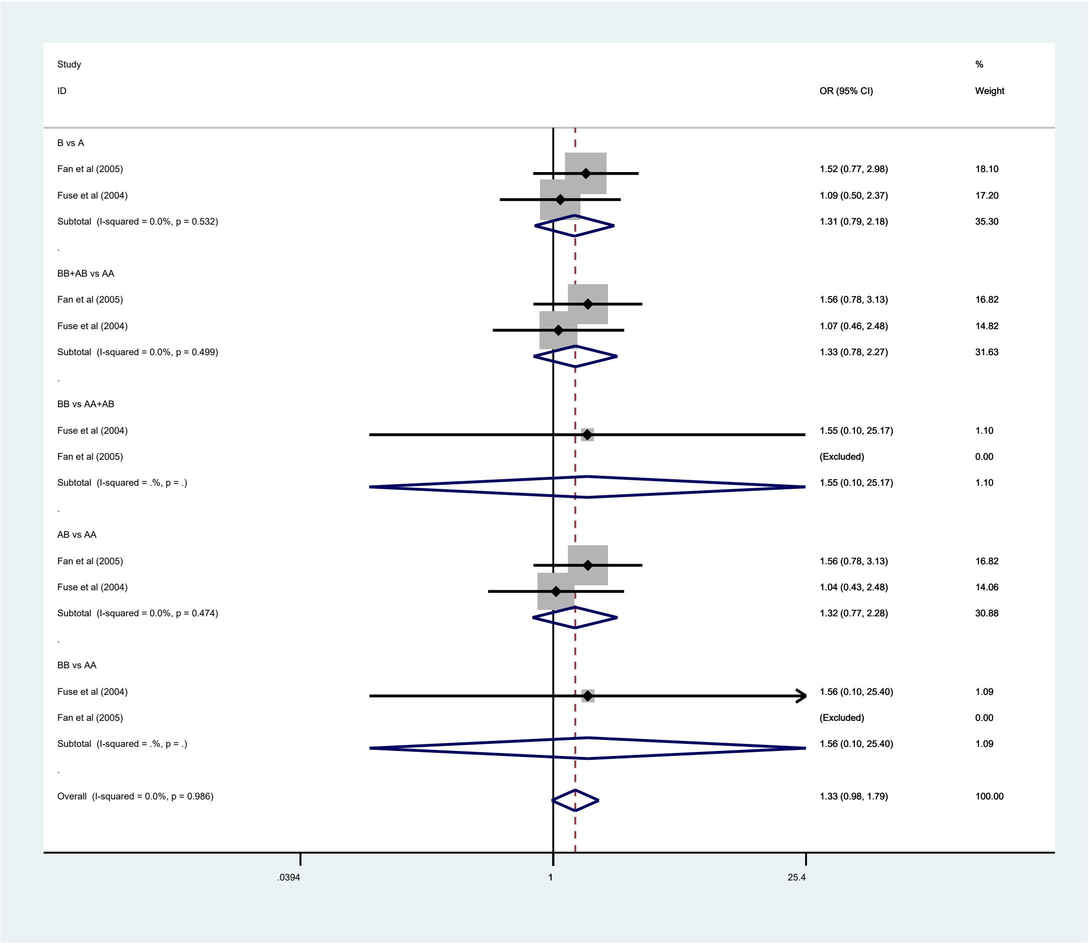

E) Forest plots demonstrating no association between *OPTN* (IVS6-10G>A) and NTG in allele (B vs A), dominant (BB+AB vs AA), recessive (BB vs AA+AB) or co-dominant (AB vs AA & BB vs AA) models respectively.

OR: odds ratio; CI: confidence interval; NTG: normal tension glaucoma.

IVS7+24G>A

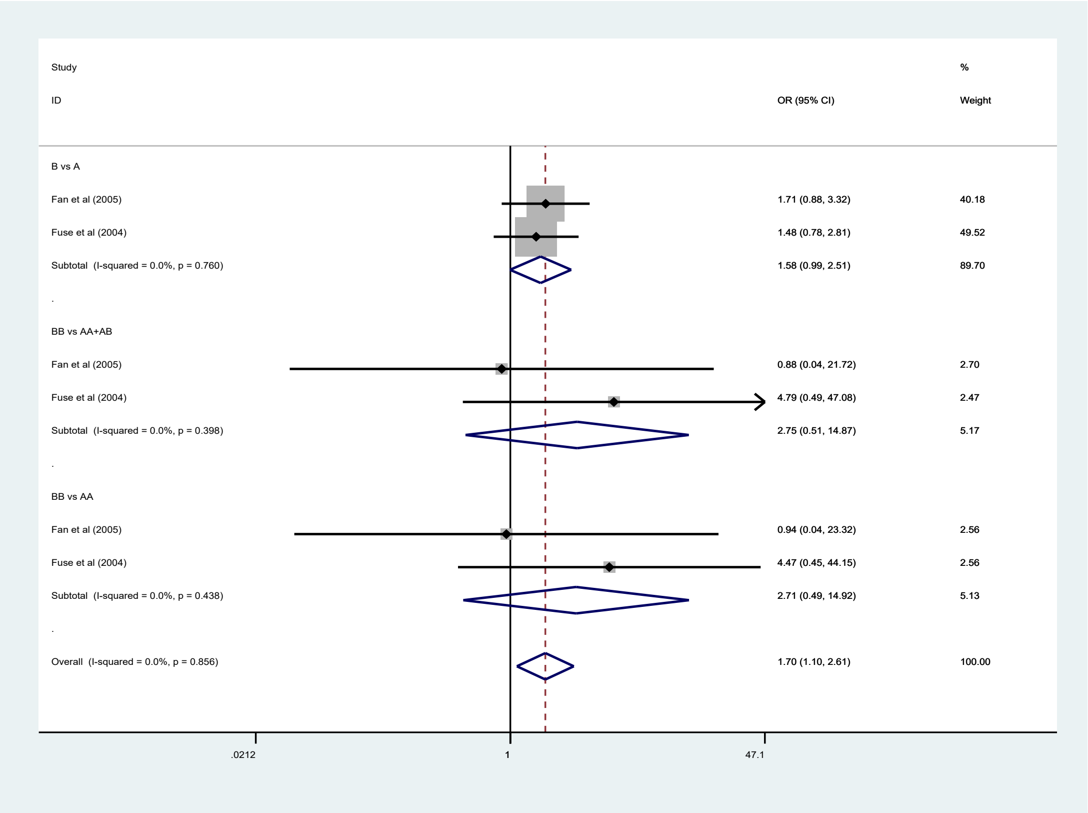

F) Forest plots demonstrating no association between *OPTN* (IVS7+24G>A) and NTG in allele (B vs A), recessive (BB vs AA+AB) or homozygote (BB vs AA) models (from fixed effects analysis). OR: odds ratio; CI: confidence interval; NTG: normal tension glaucoma.

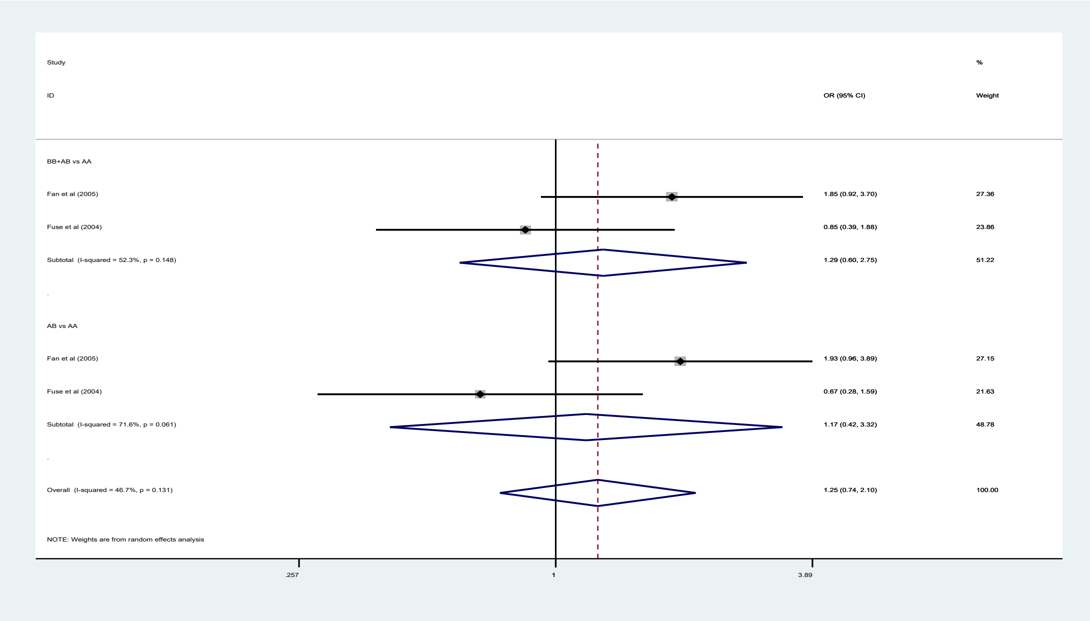

G) Forest plots demonstrating no association between *OPTN* (IVS7+24G>A) and NTG in dominant (BB+AB vs AA) or heterozygote (AB vs AA) models (from random effects analysis). OR: odds ratio; CI: confidence interval; NTG: normal tension glaucoma.

Figure S7. Associations between SNPs in *P53* gene with NTG onset.

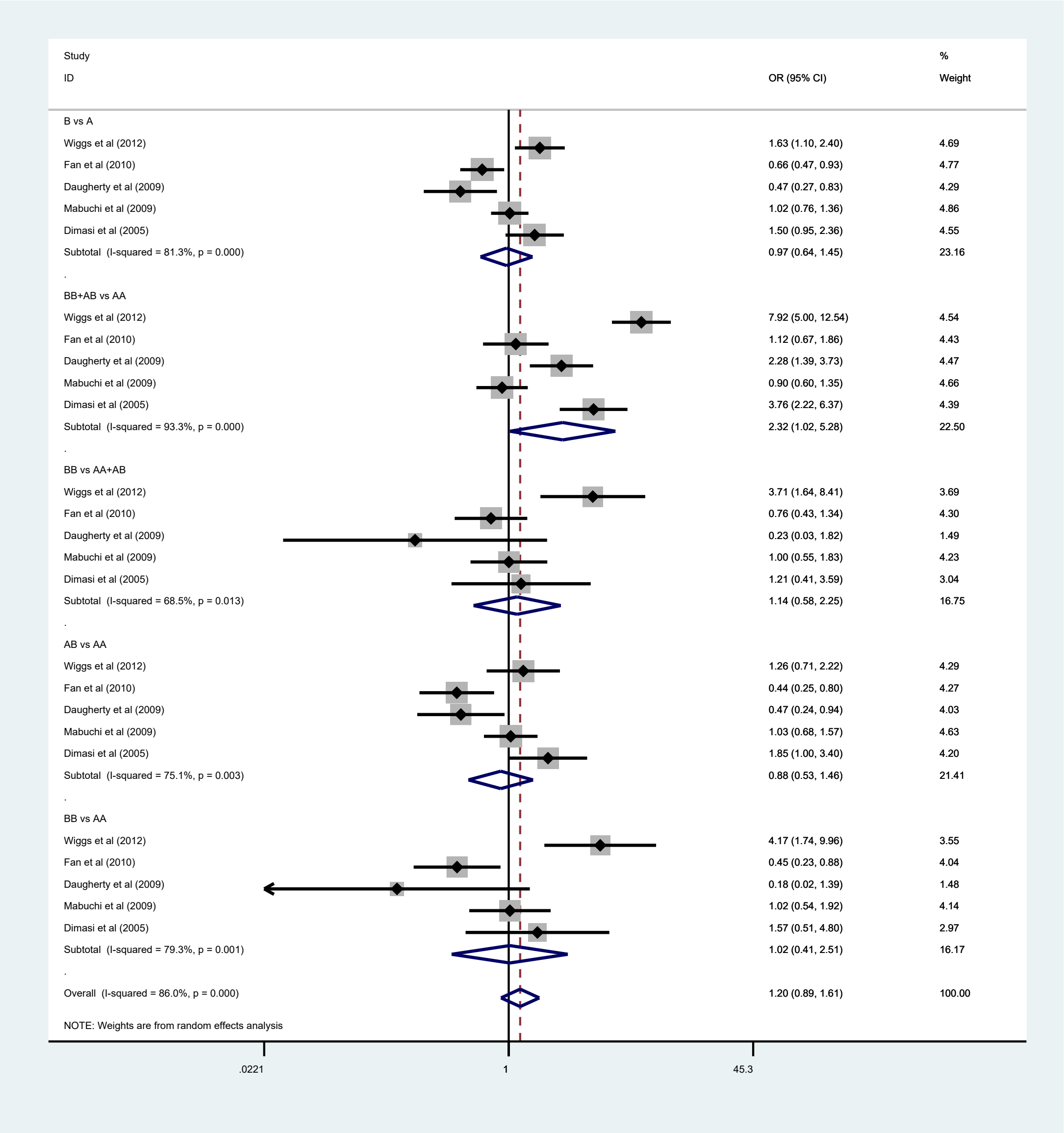

Forest plots demonstrating the association between *P53* (rs1042522) and NTG in allele (B vs A), dominant (BB+AB vs AA), recessive (BB vs AA+AB) and co-dominant (AB vs AA & BB vs AA) models respectively.

OR: odds ratio; CI: confidence interval; NTG: normal tension glaucoma.

Figure S8. Associations between SNPs in *SRBD1* gene with NTG onset.

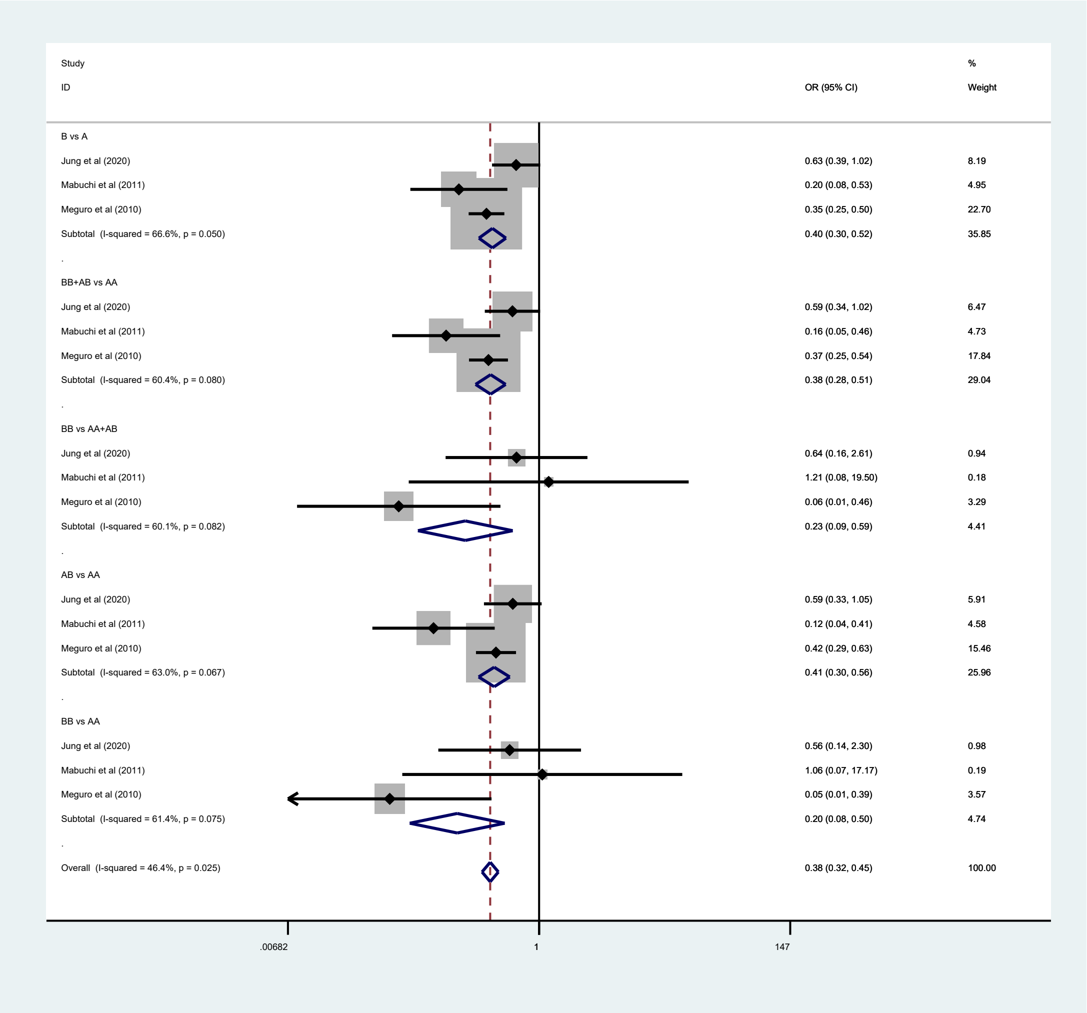

Forest plots demonstrating the association between *SRBD1* (rs3213787) and NTG in allele (B vs A), dominant (BB+AB vs AA), recessive (BB vs AA+AB) and co-dominant (AB vs AA & BB vs AA) models respectively.

OR: odds ratio; CI: confidence interval; NTG: normal tension glaucoma.

Figure S9. Associations between SNPs in *TLR4* gene with NTG onset.

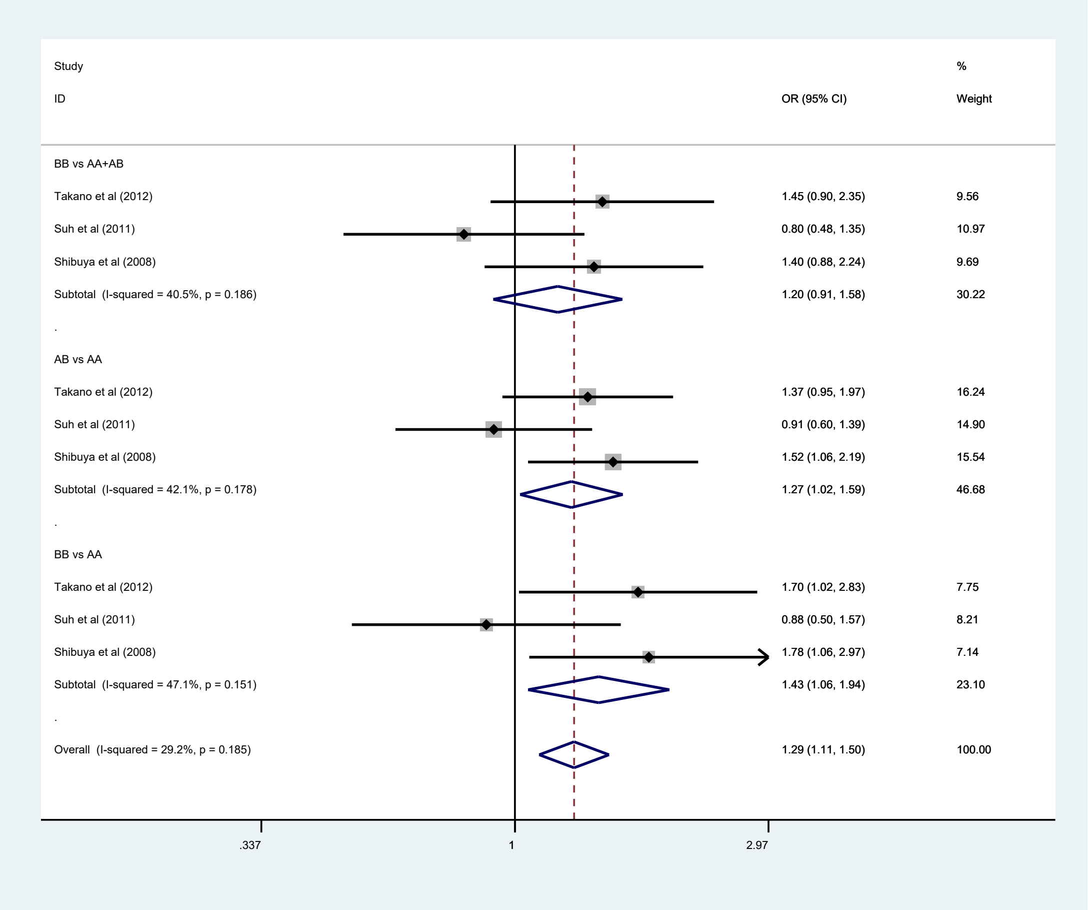

A) Forest plots demonstrating the association between TLR4 (rs10759930) and NTG in heterozygote (AB vs AA) and homozygote (BB vs AA) models (from fixed effects analysis).
OR: odds ratio; CI: confidence interval; NTG: normal tension glaucoma.

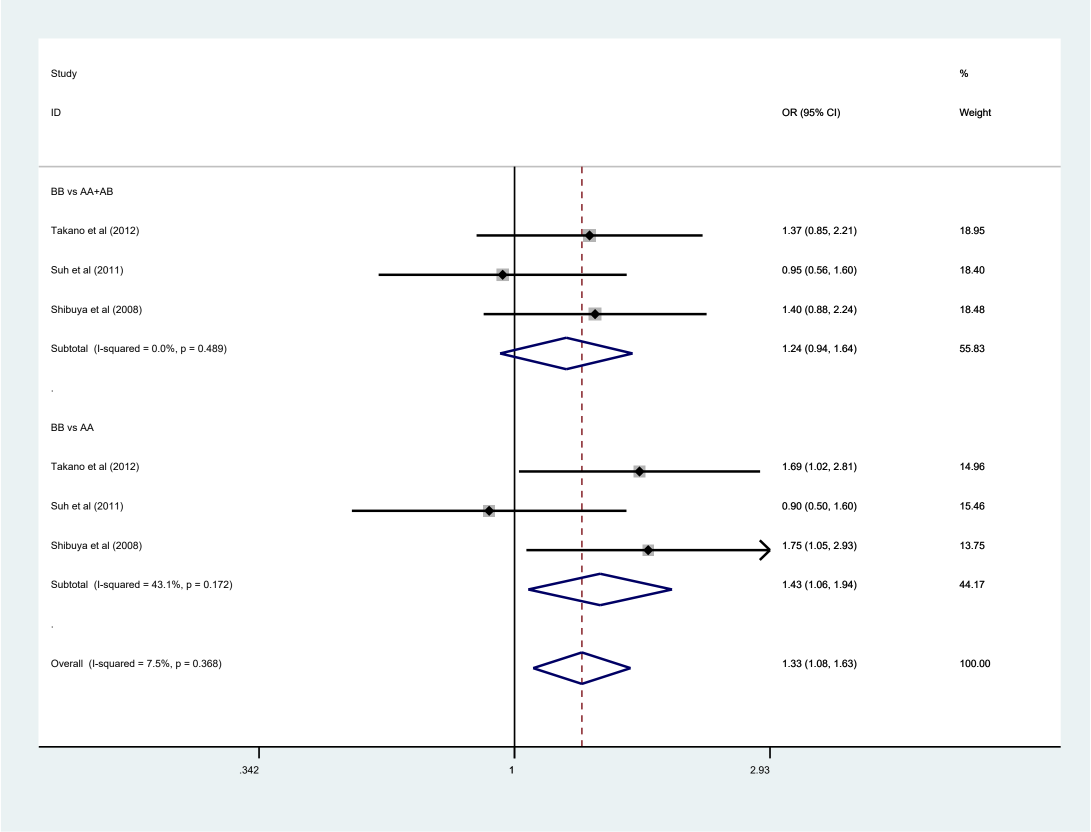

B) Forest plots demonstrating the association between TLR4 (rs1927914) and NTG in homozygote (BB vs AA) model (from fixed effects analysis).
OR: odds ratio; CI: confidence interval; NTG: normal tension glaucoma.

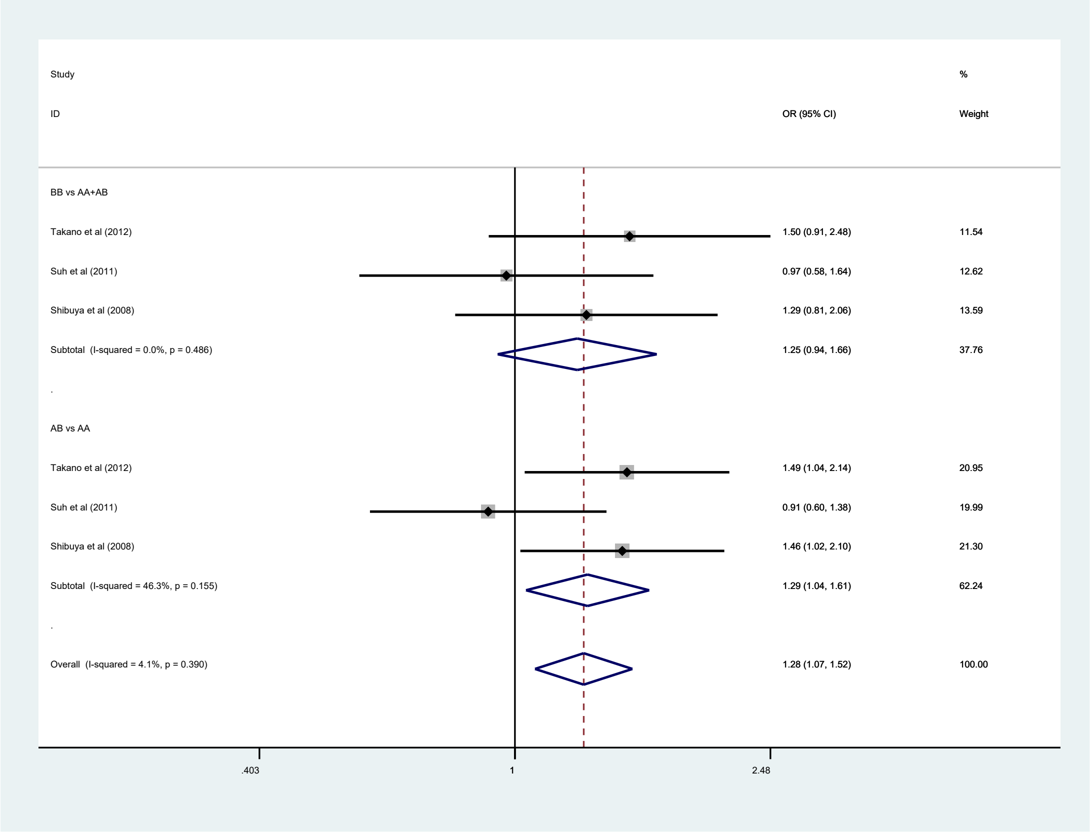

C) Forest plots demonstrating the association between TLR4 (rs1927911) and NTG in heterozygote (AB vs AA) model (from fixed effects analysis).
OR: odds ratio; CI: confidence interval; NTG: normal tension glaucoma.

rs12377632

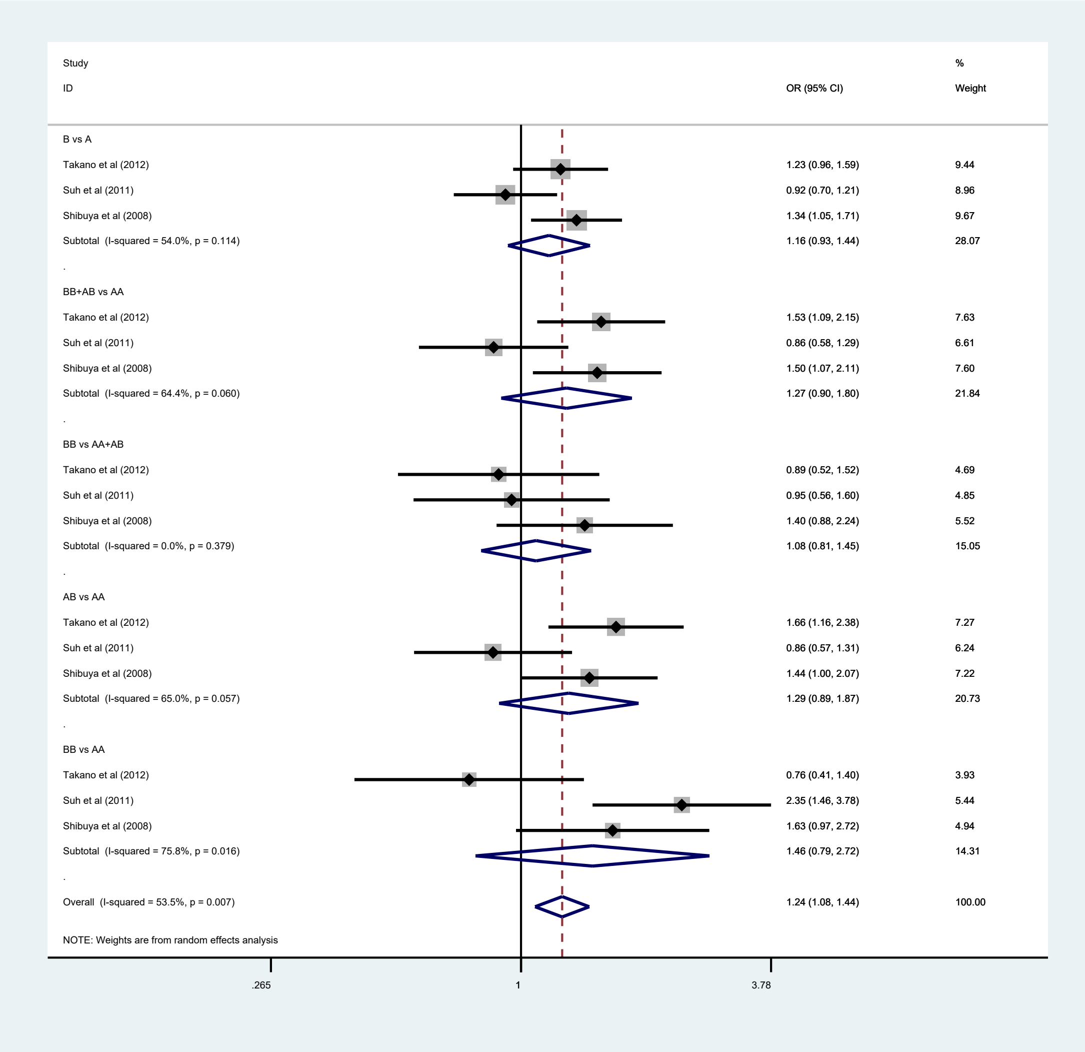

D) Forest plots demonstrating no association between *TLR4* (rs12377632) and NTG in allele (B vs A), dominant (BB+AB vs AA), recessive (BB vs AA+AB) or co-dominant (AB vs AA & BB vs AA) models respectively.
OR: odds ratio; CI: confidence interval; NTG: normal tension glaucoma.

rs2149356

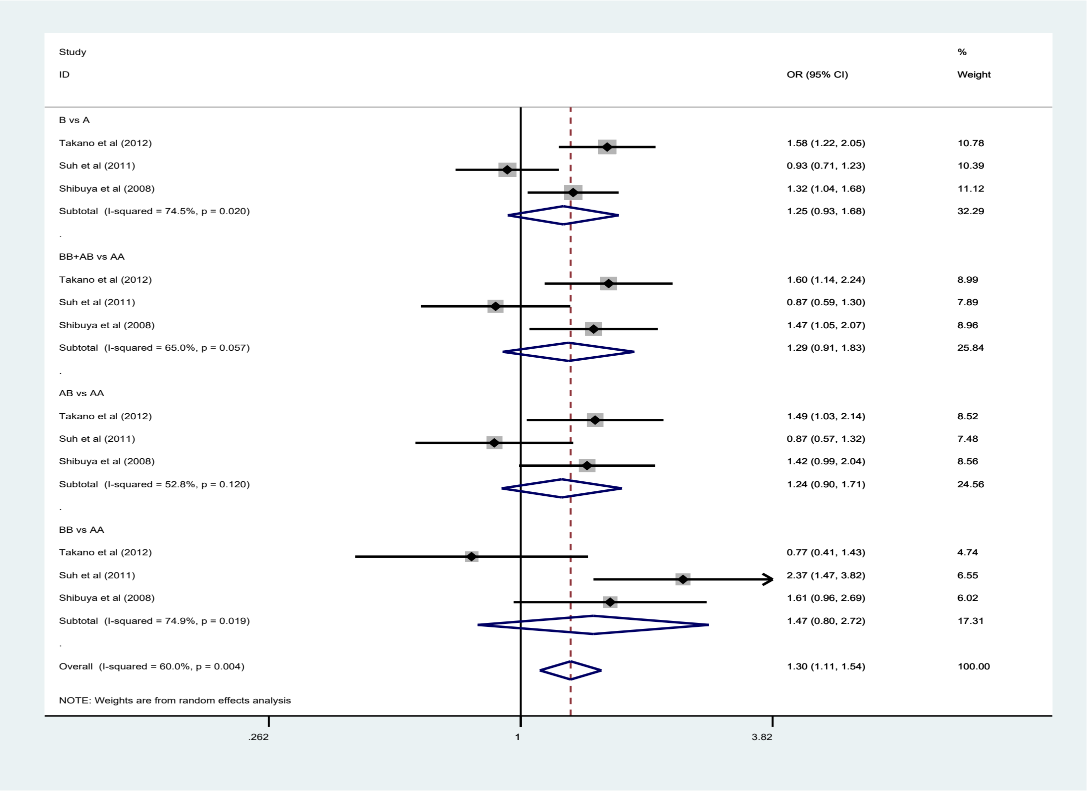

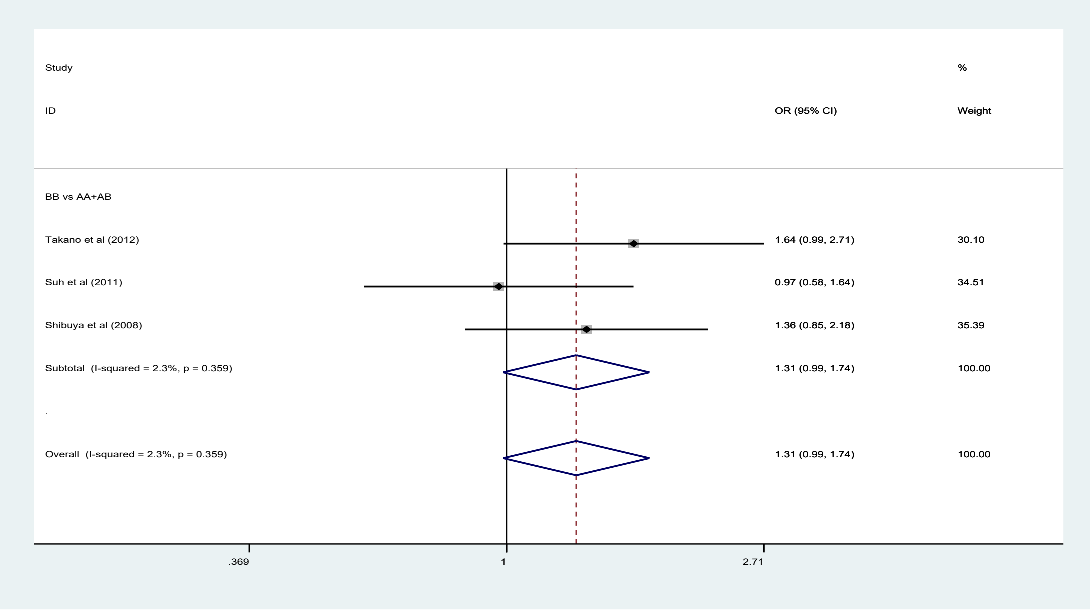

E) Forest plots demonstrating no association between *TLR4* (rs2149356) and NTG in allele (B vs A), dominant (BB+AB vs AA), heterozygote (AB vs AA), homozygote (BB vs AA) models (from random effects analysis), or recessive (BB vs AA+AB) model (from fixed effects analysis). OR: odds ratio; CI: confidence interval; NTG: normal tension glaucoma.

rs11536889

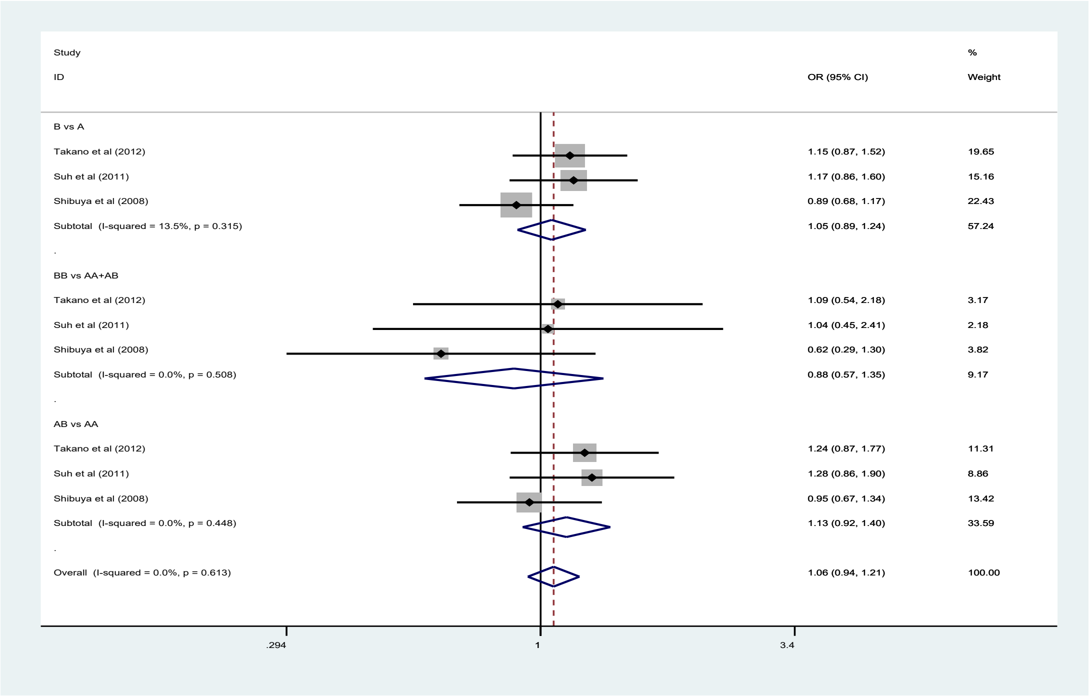

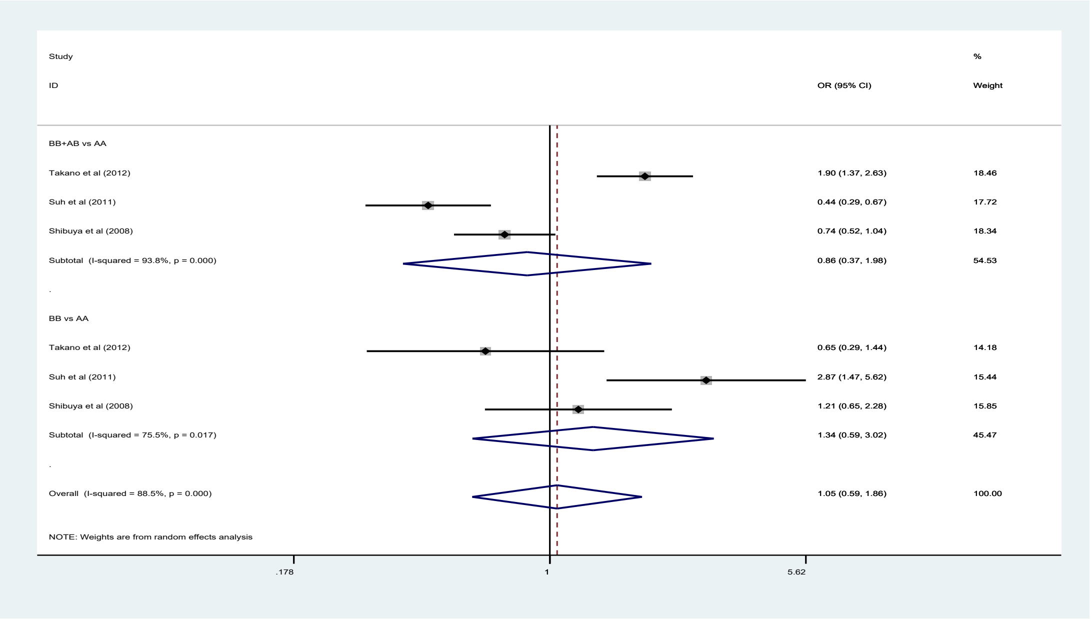

F) Forest plots demonstrating no association between *TLR4* (rs11536889) and NTG in allele (B vs A), recessive (BB vs AA+AB), heterozygote (AB vs AA) models (from fixed effects analysis), or dominant (BB+AB vs AA), homozygote (BB vs AA) models (from random effects analysis). OR: odds ratio; CI: confidence interval; NTG: normal tension glaucoma.

rs7037117

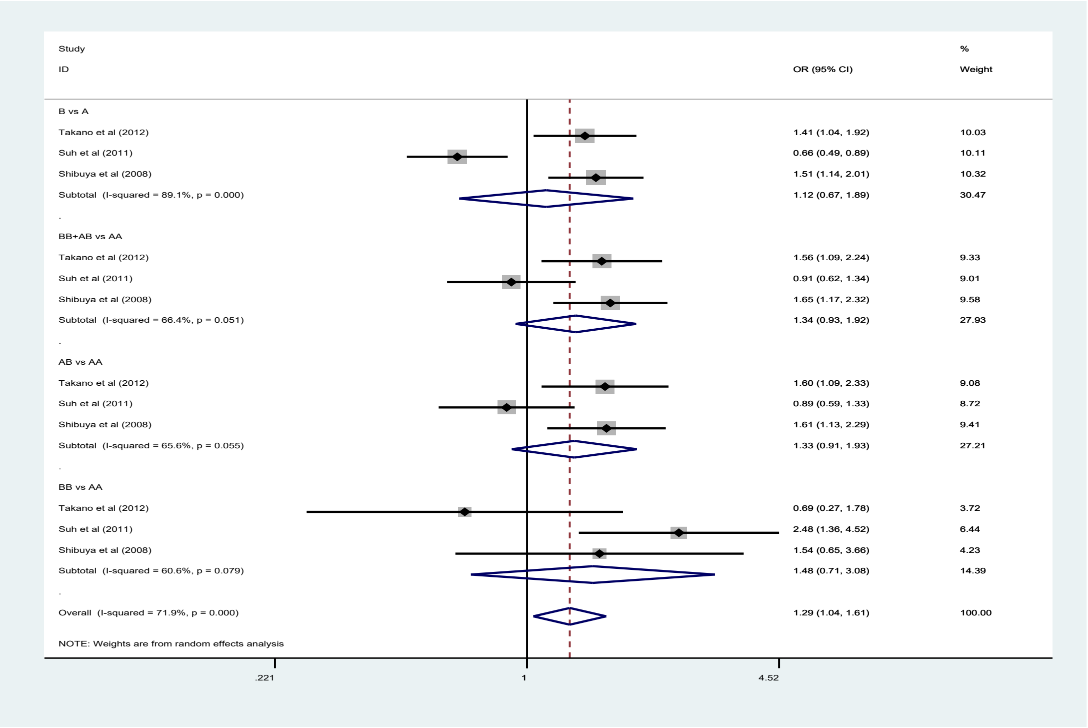

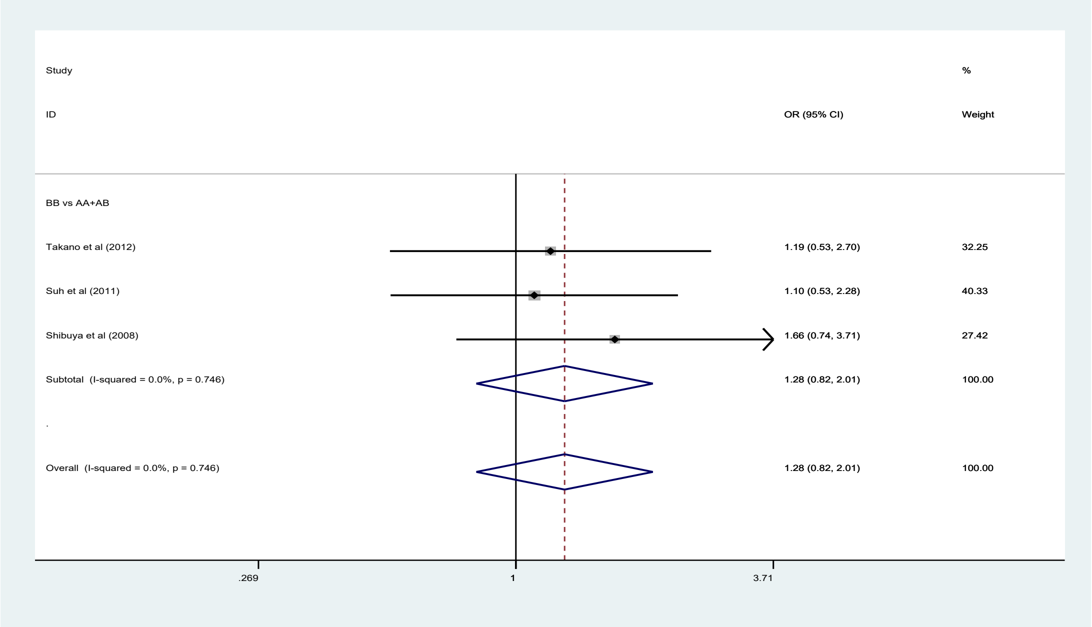

G) Forest plots demonstrating no association between *TLR4* (rs7037117) and NTG in allele (B vs A), dominant (BB+AB vs AA), heterozygote (AB vs AA), homozygote (BB vs AA) models (from random effects analysis), or recessive (BB vs AA+AB) model (from fixed effects analysis).
OR: odds ratio; CI: confidence interval; NTG: normal tension glaucoma.

rs7045953

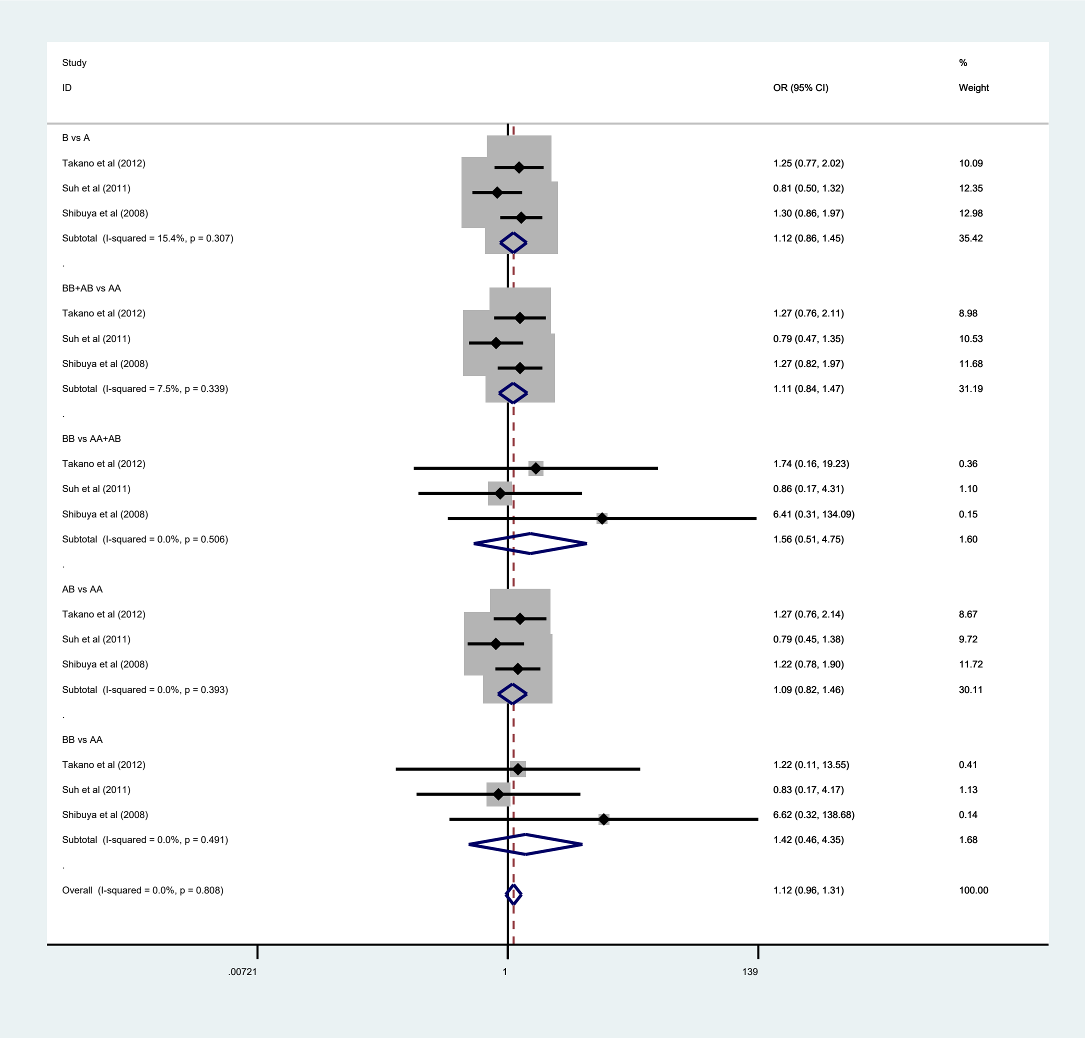

H) Forest plots demonstrating no association between *TLR4* (rs7045953) and NTG in allele (B vs A), dominant (BB+AB vs AA), recessive (BB vs AA+AB) or co-dominant (AB vs AA & BB vs AA) models respectively.

OR: odds ratio; CI: confidence interval; NTG: normal tension glaucoma.

Figure S10. Associations between SNPs in *SIX1-SIX6* gene with NTG onset.

rs10483727

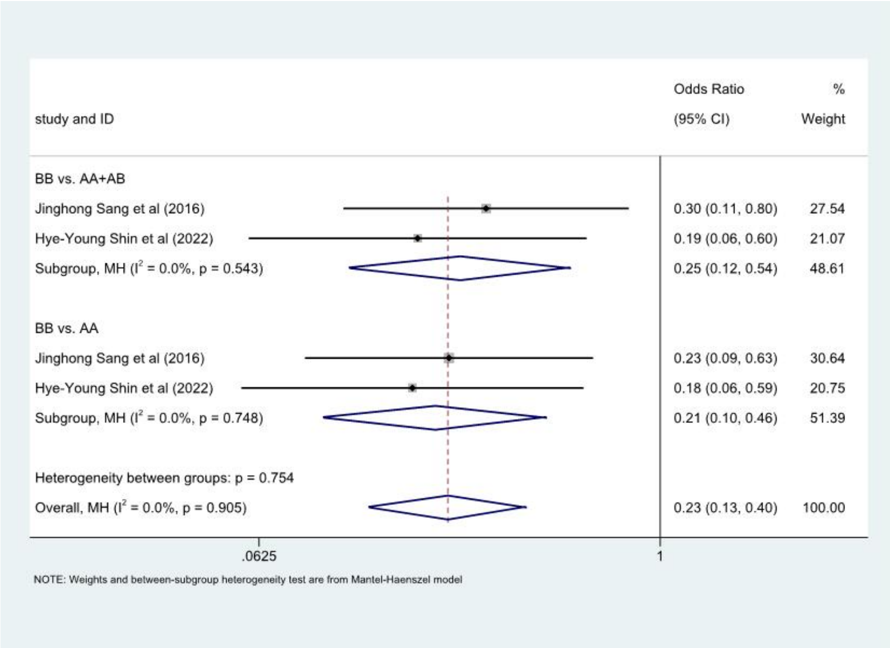

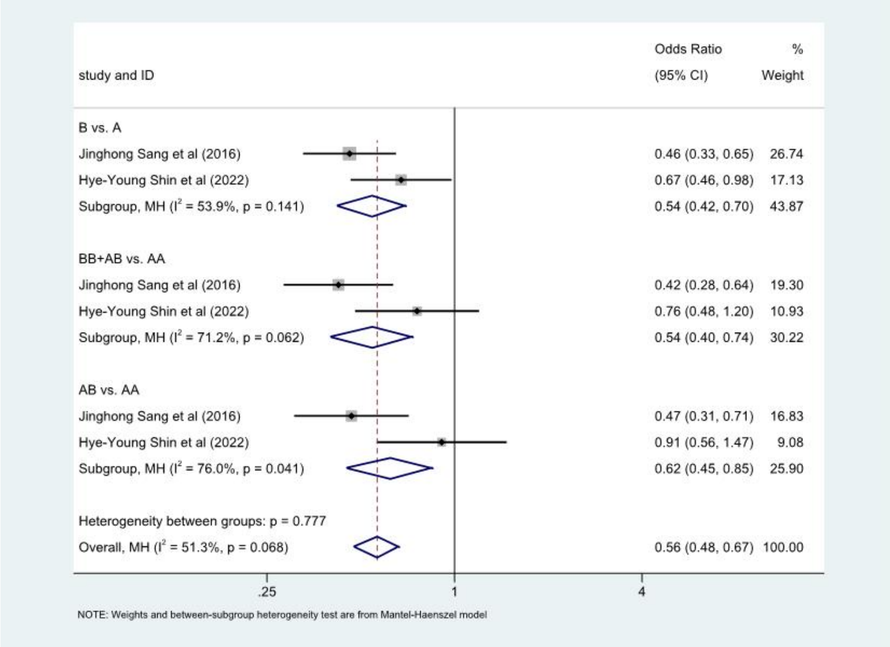

A) Forest plots exhibiting the association between *SIX1-SIX6* (rs10483727) and NTG in all models from fixed or random effects analysis respectively.

OR: odds ratio; CI: confidence interval; NTG: normal tension glaucoma.

rs33912345

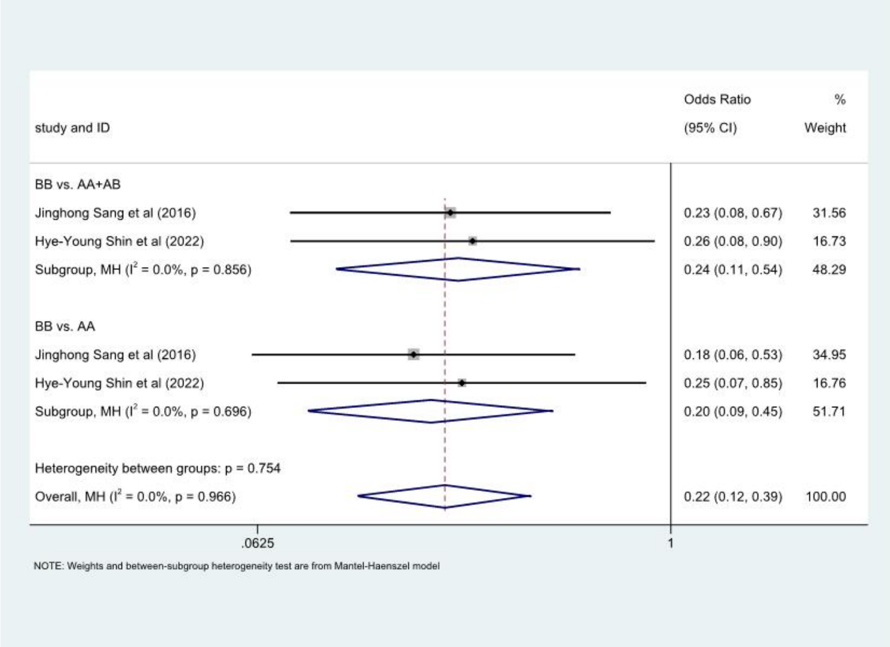

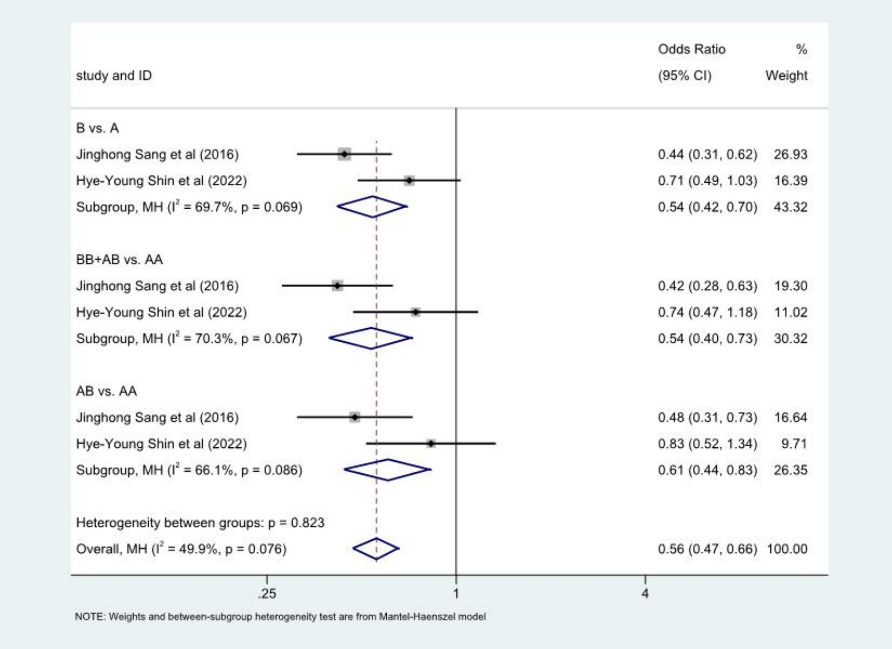

B) Forest plots exhibiting the association between *SIX1-SIX6* (rs33912345) and NTG in all models from fixed or random effects analysis respectively.

OR: odds ratio; CI: confidence interval; NTG: normal tension glaucoma.

Figure S11. Sensitivity analysis for rs7037117 in *TLR4* gene.

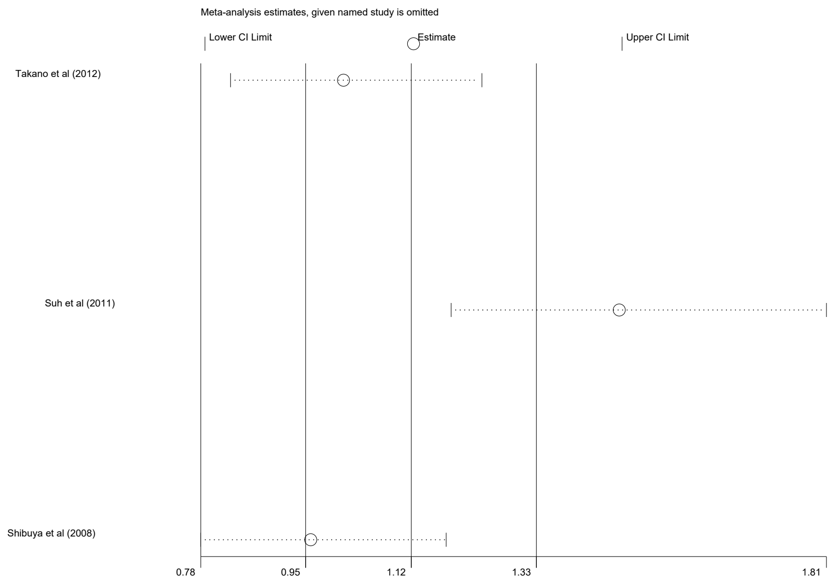

In the sensitivity analysis, the confidence interval of Suh's study was beyond the upper limit so that it was excluded for next calculation.

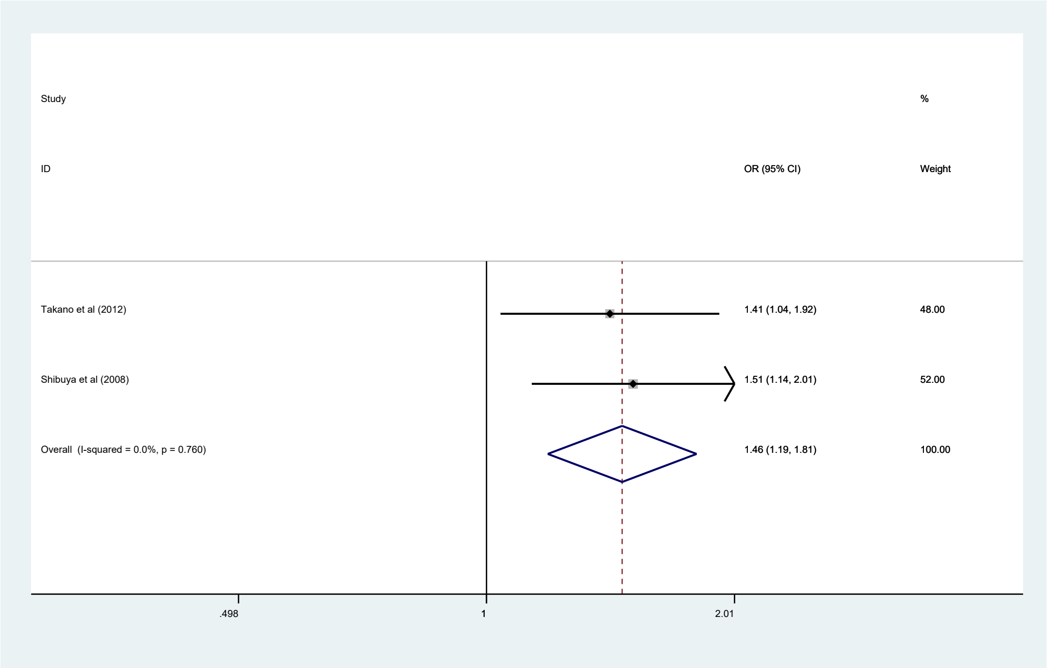

Forest plots demonstrating the significant association between *TLR4* (rs7037117) and NTG in allele (B vs A) model after one of the studies was excluded.

OR: odds ratio; CI: confidence interval; NTG: normal tension glaucoma.
