# Supplementary material for: Association of Gene Polymorphisms with Normal Tension Glaucoma: A Systematic Review and Meta-Analysis
Source: Genes (Basel). 2024 Apr 14;15(4):491. doi: 10.3390/genes15040491 (PMC11050218; doi:10.3390/genes15040491)
Supplement: Supplementary file 1 [file genes-15-00491-s001.zip › Table S1.pdf]

Table S1. Genotype frequencies for candidate SNPs in the involved studies.

Gene		SNP	Minor Allele	Study (author& year)	Genotype frequency						MAF	
					cases			controls				
Name	Symbol				AA	AB	BB	AA	AB	BB	cases	controls
apolipoprotein E	*APOE*	-491A>T	T	Lam et al., 2006	102	3	1	286	13	1	0.024	0.025
				Fan et al., 2005	102	3	1	267	13	1	0.024	0.027
		-427T>C	C	Lam et al., 2006	105	1	0	295	6	0	0.005	0.010
				Fan et al., 2005	105	1	0	276	5	0	0.005	0.009
		-219T>G	G	Lam et al., 2006	51	41	14	130	145	25	0.325	0.325
				Fan et al., 2005	51	41	14	118	139	24	0.325	0.333
endothelin receptor type A	*EDNRA*	c.-231G>A	A	Kosior-Jarecka et al., 2016	83	6	71	81	15	69	0.463	0.464
				Kim et al., 2006	29	32	6	44	39	17	0.328	0.365
		c.*70C>G	G	Kosior-Jarecka et al., 2016	27	44	89	48	51	63	0.694	0.546
				Kim et al., 2006	15	38	14	24	46	30	0.493	0.530
		c.*1222C>T	C	Kosior-Jarecka et al., 2016	79	24	56	71	39	55	0.428	0.452
			T	Kim et al., 2006	31	26	10	49	46	5	0.343	0.280
elongation of long-chain fatty acids family member 5	*ELOVL5*	rs735860	C	Mabuchi et al., 2011	51	84	23	71	89	31	0.411	0.395
				Meguro et al., 2010	51	138	116	100	162	93	0.662	0.490
hexokinase 2	*HK2*	rs678350	G	Jung et al., 2019	70	68	16	62	31	8	0.325	0.222
				Shi et al., 2013	133	113	40	161	89	21	0.337	0.257
non-catalytic region of tyrosine kinase adaptor	*NCK2*	rs2033008	A	Jung et al., 2019	78	62	14	44	41	16	0.292	0.361
				Shi et al., 2013	159	111	16	130	105	36	0.250	0.327
5,10-methylenetetrahydro-folate reductase	*MTHFR*	rs397507444, c.677 C/T	T	Clement et al., 2009	21	11	2	25	14	3	0.221	0.238
				Woo et al., 2009	25	34	19	31	50	19	0.462	0.443
				Mabuchi et al., 2006	54	58	19	48	39	19	0.366	0.363
		rs1217691063, c.1298 A/C	C	Woo et al., 2009	57	19	2	75	22	3	0.147	0.140
				Mabuchi et al., 2006	80	51	0	61	44	1	0.195	0.217
NO synthase 3	*NOS3*	rs1799983, 894 G>T	T	Jeoung et al., 2017	211	39	1	212	33	0	0.082	0.067
				Fan et al., 2010	82	16	1	157	43	1	0.091	0.112
		rs2070744, -786T>C	C	Jeoung et al., 2017	196	52	3	196	49	0	0.116	0.100
				Fan et al., 2010	81	16	2	157	40	4	0.101	0.119
Optic atrophy 1	*OPA1*	rs166850, IVS8+4C¬T	T	Milanowski et al., 2021	121	70	0	168	79	6	0.183	0.180
				Yu-Wai-Man et al., 2010	41	26	3	59	13	3	0.021	0.127

Gene		SNP	Minor Allele	Study (author& year)	Genotype frequency						MAF	
					cases			controls				
Name	Symbol				AA	AB	BB	AA	AB	BB	cases	controls
				Fan et al., 2010	89	9	1	173	27	1	0.056	0.072
				Mabuchi et al., 2007	190	4	0	182	3	0	0.010	0.008
				Yao et al., 2006	58	3	0	46	2	0	0.025	0.021
				Woo et al., 2004	62	3	0	101	0	0	0.023	0.000
				Powell et al., 2003	41	16	4	111	53	4	0.197	0.182
				Aung et al., 2002 (cohort 1)	57	26	0	86	14	0	0.157	0.070
				Aung et al., 2002 (cohort 2)	56	24	0	77	9	0	0.150	0.052
		rs10451941, IVS8+32T¬C	C	Milanowski et al., 2021	59	97	75	98	110	48	0.535	0.402
				Yu-Wai-Man et al., 2014	11	37	22	21	37	17	0.579	0.473
				Fan et al., 2010	38	49	12	74	104	23	0.369	0.373
				Mabuchi et al., 2007	125	63	6	146	35	4	0.193	0.116
				Yao et al., 2006	14	30	17	16	26	6	0.525	0.396
				Woo et al., 2004	40	18	7	68	29	4	0.246	0.183
				Powell et al., 2003	17	27	17	43	104	21	0.500	0.435
				Aung et al., 2002 (cohort 1)	47	36	0	72	28	0	0.217	0.140
				Aung et al., 2002 (cohort 2)	41	39	0	55	31	0	0.244	0.180
optineurin	*OPTN*	c.603T>A, Met98Lys	A	Fan et al., 2005	67	36	3	200	74	7	0.198	0.157
				Funayama et al., 2004	169	43	5	182	35	1	0.122	0.085
				Fuse et al., 2004	55	9	1	95	5	0	0.085	0.025
		c.412G>A, Thr34Thr	A	Fan et al., 2005	66	34	6	222	57	2	0.217	0.109
				Funayama et al., 2004	148	62	7	166	50	2	0.175	0.124
				Fuse et al., 2004	37	27	1	65	33	2	0.223	0.185
		IVS6-5T>C	C	Fan et al., 2005	80	20	6	229	48	4	0.151	0.091
				Fuse et al., 2004	38	23	4	52	42	6	0.246	0.270
		IVS6-10G>A	A	Fan et al., 2005	92	14	0	256	25	0	0.066	0.044
				Fuse et al., 2004	54	10	1	84	15	1	0.092	0.085
		IVS7+24G>A	A	Fan et al., 2005	91	15	0	258	22	1	0.071	0.043
				Fuse et al., 2004	53	9	3	79	20	1	0.154	0.150
tumor protein p53	*p53*	rs1042522, -Arg72Pro	C	Wiggs et al., 2012	25	29	10	198	183	19	0.383	0.276
				Fan et al.,2010	34	43	22	38	108	55	0.439	0.542
				Daugherty et al., 2009	36	15	1	82	72	13	0.163	0.293

Gene		SNP	Minor Allele	Study (author& year)	Genotype frequency						MAF	
					cases			controls				
Name	Symbol				AA	AB	BB	AA	AB	BB	cases	controls
				Mabuchi et al., 2009	92	95	26	83	83	23	0.345	0.341
				Dimasi et al., 2005	29	28	5	109	57	12	0.306	0.228
S1 RNA binding domain 1	*SRBD1*	rs3213787	G	Jung et al., 2020	260	44	1	241	96	18	0.129	0.189
				Mabuchi et al., 2011	122	33	4	68	31	4	0.016	0.073
				Meguro et al., 2010	154	3	1	164	26	1	0.075	0.188
Toll-like receptor 4	*TLR4*	rs10759930	C	TAKANO et al., 2012	141	159	65	103	85	28	0.396	0.326
				Suh et al., 2011	52	72	23	126	191	63	0.401	0.415
				Shibuya et al., 2008	81	127	42	137	141	40	0.422	0.347
		rs1927914	G	TAKANO et al., 2012	137	164	64	105	82	29	0.456	0.324
				Suh et al., 2011	52	72	23	126	192	62	0.388	0.415
				Shibuya et al., 2008	82	126	42	137	141	40	0.420	0.347
		rs1927911	A	TAKANO et al., 2012	139	166	60	106	85	25	0.392	0.254
				Suh et al., 2011	53	71	23	129	190	61	0.398	0.411
				Shibuya et al., 2008	87	122	41	141	135	42	0.408	0.344
		rs12377632	T	TAKANO et al., 2012	137	190	38	104	87	25	0.364	0.317
				Suh et al., 2011	54	70	23	127	191	62	0.395	0.414
				Shibuya et al., 2008	86	122	42	140	138	40	0.412	0.343
		rs2149356	T	TAKANO et al., 2012	139	164	62	107	85	24	0.395	0.291
				Suh et al., 2011	54	70	23	128	191	61	0.395	0.412
				Shibuya et al., 2008	87	122	41	140	138	40	0.408	0.343
		rs11536889	C	TAKANO et al., 2012	196	145	24	127	76	13	0.264	0.238
				Suh et al., 2011	77	62	8	221	139	20	0.265	0.236
				Shibuya et al., 2008	146	93	11	177	119	22	0.235	0.256
		rs7037117	G	TAKANO et al., 2012	222	125	18	153	54	9	0.221	0.167
				Suh et al., 2011	85	51	11	211	143	26	0.185	0.257
				Shibuya et al., 2008	138	98	14	213	94	11	0.252	0.182
		rs7045953	G	TAKANO et al., 2012	313	50	2	191	24	1	0.074	0.060
				Suh et al., 2011	126	19	2	314	60	6	0.078	0.095
				Shibuya et al., 2008	203	45	2	269	49	0	0.098	0.077
WD repeat domain 36	*WDR36*	rs17553936, IVS16–30A>G	G	Fan et al., 2009	27	13	2	47	27	3	0.202	0.214
				Miyazawa et al., 2007	76	25	2	72	41	5	0.141	0.216

Gene		SNP	Minor Allele	Study (author& year)	Genotype frequency						MAF	
					cases			controls				
Name	Symbol				AA	AB	BB	AA	AB	BB	cases	controls
sine oculis homeobox homolog 1- sine oculis homeobox homolog 6	*SIX1-SIX6*	rs10483727	C	Shin et al., 2022	129	77	4	64	42	11	0.202	0.274
				Sang et al., 2016	131	45	5	140	103	23	0.152	0.280
		rs33912345	A	Shin et al., 2022	130	76	4	64	45	8	0.200	0.261
				Sang et al., 2016	132	45	4	141	101	24	0.146	0.280

SNP: single nucleotide polymorphism; MAF: minor allele frequency.
