# Supplementary material for: Association of Gene Polymorphisms with Normal Tension Glaucoma: A Systematic Review and Meta-Analysis
Source: Genes (Basel). 2024 Apr 14;15(4):491. doi: 10.3390/genes15040491 (PMC11050218; doi:10.3390/genes15040491)
Supplement: Supplementary file 1 [file genes-15-00491-s001.zip › Table S2.pdf]

Table S2. Genetic associations of NTG in different ethnicities.

No.	Gene	SNP	Genetic model	Pooled sample size		Fixed or random effect model*		Heterogenity test	
				cases	controls	OR (95%CI)	*p*	*p* (Q)	*I²* (%)
**Asian**									
1	*MTHFR*	rs397507444	B vs. A	209	206	1.04 (0.79-1.38)	0.772	0.830	0.0
			BB+AB vs. AA	209	206	1.08 (0.73-1.62)	0.694	0.609	0.0
			BB vs. AA+AB	209	206	1.02 (0.62-1.68)	0.930	0.264	19.8
			AB vs. AA	209	206	1.10 (0.72-1.70)	0.658	0.319	0.0
			BB vs. AA	209	206	1.03 (0.59-1.79)	0.911	0.558	0.0
2	*OPA1*	rs166850	B vs. A	358	487	1.01 (0.56-1.84)	0.964	0.199	38.0
			BB+AB vs. AA	358	487	0.98 (0.53-1.83)	0.951	0.174	42.8
			BB vs. AA+AB	99	201	2.04 (0.13-32.97)	0.615	NA	0.0
			AB vs. AA	358	487	0.95 (0.50-1.80)	0.873	0.157	45.9
			BB vs. AA	99	201	1.94 (0.12-31.44)	0.640	NA	100.0
3	*OPA1*	rs10451941	B vs. A	358	487	1.36 (0.91-2.02)	0.136	0.071	62.1
			BB+AB vs. AA	358	487	1.37 (0.83-2.26)	0.217	0.069	62.7
			BB vs. AA+AB	358	487	1.40 (0.80-2.46)	0.241	0.405	0.0
			AB vs. AA	358	487	1.30 (0.74-2.26)	0.360	0.052	66.2
			BB vs. AA	358	487	1.45 (0.81-2.61)	0.215	0.361	1.8
4	*p53*	rs1042522	B vs. A	312	390	0.83 (0.54-1.26)	0.380	0.061	71.5
			BB+AB vs. AA	312	390	0.98 (0.71-1.34)	0.894	0.518	0.0
			BB vs. AA+AB	312	390	0.86 (0.57-1.30)	0.484	0.505	0.0
			AB vs. AA	312	390	0.70 (0.31-1.58)	0.387	0.021	81.1
			BB vs. AA	312	390	0.68 (0.30-1.53)	0.352	0.082	67.0
**Caucasian**									
1	*OPA1*	rs166850	B vs. A	485	682	**1.65 (1.06-2.57)**	**0.026**	0.016	67.1
			BB+AB vs. AA	485	682	**2.39 (1.15-4.96)**	**0.019**	0.000	84.8
			BB vs. AA+AB	322	496	0.97 (0.18-5.21)	0.967	0.087	59.0
			AB vs. AA	485	682	**2.21 (1.09-4.46)**	**0.027**	0.000	82.2
			BB vs. AA	322	496	1.09 (0.22-5.47)	0.916	0.109	54.8
2	*OPA1*	rs10451941	B vs. A	525	685	**1.57 (1.32-1.87)**	**0.000**	0.852	0.0
			BB+AB vs. AA	525	685	**1.66 (1.29-2.13)**	**0.000**	0.376	5.3
			BB vs. AA+AB	362	499	**2.04 (1.47-2.82)**	**0.000**	0.500	0.0
			AB vs. AA	525	685	**1.46 (1.12-1.90)**	**0.005**	0.168	38.0
			BB vs. AA	362	499	**2.45 (1.67-3.61)**	**0.000**	0.894	0.0

No.	Gene	SNP	Genetic model	Pooled sample size		Fixed or random effect model*		Heterogenity test	
				cases	controls	OR (95%CI)	*p*	*p* (Q)	I^2 (%)
3	*p53*	rs1042522	B vs. A	178	745	1.07 (0.53-2.17)	0.842	0.001	85.4
			BB+AB vs. AA	178	745	**4.10 (1.96-8,59)**	**0.000**	0.001	85.2
			BB vs. AA+AB	178	745	1.30 (0.32-5.26)	0.716	0.019	74.8
			AB vs. AA	178	745	1.05 (0.49-2.22)	0.903	0.012	77.4
			BB vs. AA	178	745	1.33 (0.28-6.31)	0.719	0.010	78.2

* If p (Q) was ≥0.05 and I^2 value <50%, the fixed-effect model was chosen, otherwise a random-effect model was used.

NTG: normal tension glaucoma; SNP: single nucleotide polymorphism; OR: odds ratio; CI: confidence interval; *MTHFR:* 5,10-methylenetetrahydro-folate reductase; *OPA1*: Optic atrophy 1.
